# XSE-TomatoNet: An explainable AI based tomato leaf disease classification method using EfficientNetB0 with squeeze-and-excitation blocks and multi-scale feature fusion

**DOI:** 10.1016/j.mex.2025.103159

**Published:** 2025-01-06

**Authors:** Md Assaduzzaman, Prayma Bishshash, Md. Asraful Sharker Nirob, Ahmed Al Marouf, Jon G. Rokne, Reda Alhajj

**Affiliations:** a Department of Computer Science and Engineering, Daffodil International University, Daffodil Smart City, Birulia 1216, Dhaka, Bangladesh; b Department of Computer Science, University of Calgary, Alberta, T2N 1N4, Canada; c Department of Computer Engineering, Istanbul Medipol University, Istanbul 34810, Turkiye; d Department of Heath Informatics, University of Southern Denmark, 5230 Odense, Denmark

**Keywords:** Tomato Leaf diseases, EfficientNet-B0, Explainable AI, SHAP, LIME, Grad-CAM, Grad-CAM++, XSE-TomatoNet

## Abstract

Tomatoes are globally valued for their nutritional benefits and unique taste, playing a crucial role in agricultural productivity. Accurate diagnosis of tomato leaf diseases is vital to avoid ineffective treatments that can harm plants and ecosystems. While deep learning models excel in classifying these diseases, distinguishing subtle variations remains challenging. This study introduces XSE-TomatoNet, an enhanced version of EfficientNetB0, incorporating Squeeze-and-Excitation (SE) blocks and multi-scale feature fusion to boost classification performance. XSE-TomatoNet extracts multi-scale features, refines them with SE blocks, and merges them through Global Average Pooling, providing detailed and broad insights for precise disease classification. Our approach achieves an impressive accuracy of 99.11%, with 99% precision and recall, outperforming models like MobileNet and VGG19, especially when combined with data augmentation and ablation studies. The model achieved an average training accuracy of 99.41% and a validation accuracy of 98.88% in 10-fold cross-validation, showing strong generalization to unseen data. We also used LIME and SHAP for model interpretability, offering insights into the decision-making process, and employed Grad-CAM and Grad-CAM++ to visually highlight key areas in leaf images. Finally, the best model was integrated into a web-based system for practical use by tomato cultivators.

• XSE-TomatoNet is an enhanced version of EfficientNetB0 which incorporates Squeeze-and-Excitation (SE) blocks and multi-scale feature fusion.
• XSE-TomatoNet outperformed MobileNet (87.44%) and VGG-19 (95.50%), in terms of accuracy, achieving 99.41%.
• Integration of interpretation using LIME and SHAP models gives higher level understanding of the diseases and employment of Grad-CAM and Grad-CAM++ shows visual representation of the diseased leaves.

## Graphical abstract

**Figure:**
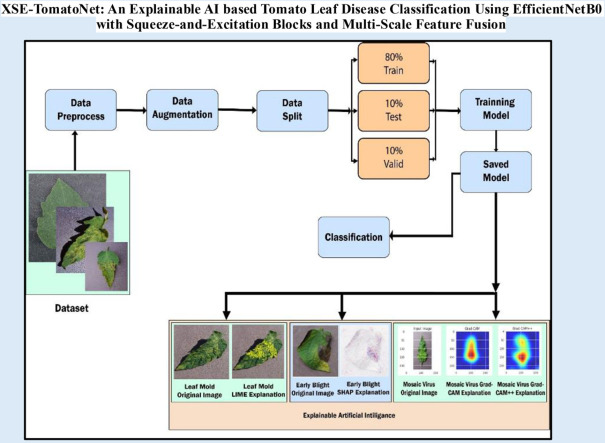

Specifications table

This table provides general information on your method.

**Table:** 

Subject area:	Computer Science
More specific subject area:	Deep Learning, computer vision, Agricultural Informatics, Plant Disease Detection, Plant Health Monitoring, Data Science in Agriculture.
Name of your method:	XSE-TomatoNet
Name and reference of original method:	1. Tan, Mingxing, and Quoc Le. “Efficientnet: Rethinking model scaling for convolutional neural networks.” In International conference on machine learning, pp. 6105–6114. PMLR, 2019. 2. T. Agrawal, P. Choudhary, A. Shankar, P. Singh, and Manoj Diwakar, “MultiFeNet: Multi‐scale feature scaling in deep neural network for the brain tumour classification in MRI images,” International journal of imaging systems and technology, vol. 34, no. 1, Aug. 2023, doi: https://doi.org/10.1002/ima.22956.
Resource availability:	https://www.kaggle.com/datasets/cookiefinder/tomato-disease-multiple-sources

## Introduction

Agriculture is the cornerstone of global economic growth and provides food and income for millions of people. To meet demand, agricultural production must grow by 70% by the year 2050, according to the Food and Agriculture Organization of the United Nations (FAO). With the help of this ambitious goal, the world's population's nutritional needs will be met and the demand for food will be reduced [1]. Agriculture is the backbone of Bangladesh's economy, considerably contributing to its Gross Domestic Product (GDP) of Tk 3223,938 million for the fiscal year 2018–19 and Tk 3476,381 million in 2019–20, according to a 2012 Bangladesh Bureau of Statistics (BBS) report. According to the BBS in 2012, the agricultural industry spans a significant region, with cultivated land comprising a total of 39,678 thousand acres [2]. Because of its broad consumption and economic significance, tomato growing is important in the agricultural world.

However, the path to optimizing tomato yield and quality is fraught with challenges, primarily due to the susceptibility of tomato plants to various diseases. Traditional methods of disease diagnosis, reliant on manual inspection, are increasingly inadequate in the face of these challenges. The subjective nature of these methods, compounded by the fatigue and error-proneness inherent in human judgment, necessitates a shift towards more efficient, accurate, and automated solutions. Transfer learning has emerged as a potent tool in deep learning, especially in tasks related to image classification. They have proven to be highly effective in identifying plant diseases [[3], [4], [5]]. However, their wide adoption is still not enough due to the opaque secrecy of deep learning models that are often termed as 'black boxes'—it does not provide reasoning or cause-effect analysis—and therefore pose a significant challenge, specifically in strategic sectors like agriculture, where high explain ability is required.

Addressing this, our study presents a state-of-the-art model that utilizes the EfficientNetB0 with Squeeze-and-Excitation Blocks and Multi-Scale Feature Fusion, for its effectiveness and computational efficiency. By conducting a thorough ablation study, we improved our model to achieve exceptional accuracy in classifying 11 different tomato leaf diseases. This study not only highlights the robustness of the model but also explores the domain of explainable AI by utilizing SHAP, LIME, Grad-CAM, and Grad-CAM++ to explain the model's decision-making process. The combination of focusing on both accuracy and interpretability paves the way for a cutting-edge diagnostic tool that is not only highly effective but also transparent and trustworthy. In addition, our proposed model is integrated into a user-friendly web application, providing a user-centric platform for farmers and agricultural stakeholders for disease diagnosis and management.

## Background

Tomatoes are highly valued and extensively cultivated in the agricultural sector, playing a vital role in the economies of numerous countries worldwide. The tomato industry faces several challenges, with diseases affecting tomato plants being a significant concern due to their cultural and economic significance. Conventional detection techniques, often relying on manual processes and susceptible to errors, prove ineffective in efficiently addressing these challenges. Deep learning algorithms have the potential to greatly influence precision agriculture, particularly in the identification of diseases. This literature review delves into research on employing deep learning techniques for the detection of diseases in tomato leaves, highlighting their advantages over traditional approaches.

Al-Shamasneh et al. [6] introduced a novel method for detecting tomato leaf diseases is presented using conformable polynomials image features. The methodology involves preprocessing, feature extraction, and classification, utilizing the Plant Village dataset for training. The extracted texture features were fed into an SVM classifier, achieving an accuracy of 98.80%. This automated system enables early detection of plant diseases, allowing for targeted interventions that reduce pesticide use and improve crop yield.

Natarajan et al. [7] proposed a deep convolutional neural network architecture for the efficient diagnosis of plant diseases, utilising explainable AI methods. The study utilized fine-tuning of a deep neural network to categorized plant diseases using the PlantVillage dataset, comprising 38 classes. The suggested method integrates a tailored K-Nearest Neighbours (KNN) algorithm for feature classification, attaining exceptional results with a validation accuracy of 99.95% and an AUC of 1. The system exhibits enhanced performance relative to other advanced deep learning methods, making it remarkably appropriate for early detection of plant diseases and beneficial in boosting food security.

Sagar et al. [8] conducted an extensive survey on plant leaf disease detection, evaluating both traditional machine learning and deep learning techniques. The study emphasizes the significance of agriculture in countries like India, where plant diseases can substantially affect yield and economic growth. The authors highlight the importance of Explainable AI (XAI) in enhancing the interpretability of deep learning models, ensuring that end-users, such as farmers and agricultural professionals, can trust and understand the decisions made by these models. By summarizing various plant disease datasets and models, the paper provides key insights for developing transparent and efficient AI solutions for combating plant diseases.

Bhandari et al. [9] proposed a comprehensive framework, BotanicX-AI, for the identification of nine tomato leaf diseases, such as bacterial spot, early blight, and Septoria leaf spot, utilising a deep learning methodology. The authors employed the EfficientNetB5 model, attaining an impressive average test accuracy of 99.07% over 10 cross-validation folds. To improve model interpretability, they integrated explainable AI (XAI) approaches, particularly GradCAM and LIME, which provided visual elucidations of the decision-making process by emphasizing the key regions of the leaf that influenced disease classification. This transparency is essential for agricultural stakeholders to trust and implement AI technologies in practical farming applications.

Paul et al. [10] highlighted the importance of early diagnosis for improved plant health in the real-time identification of leaf diseases of tomatoes using CNN. To prevent using ineffective medicines, accurate disease identification is essential. With the help of the VGG-16 and VGG-19 models, they used transfer learning to create a unique CNN. The custom CNN obtained a remarkable 95.00% accuracy and recall through data augmentation. Additionally, they have developed a comprehensive system that tomato cultivators may use through the web and Android platforms to help with disease classification.

A. Nag et al. [11] presented a novel approach for swift and intelligent detection of tomato leaf diseases using a mobile app. They optimized widely recognized CNN architectures, including AlexNet, SqueezeNet-1.1, ResNet-50, DenseNet-121, Squeeze Network-1.1, and VGG19 by employing transfer learning and CNNs on a dataset comprising tomato leaf images obtained through PlantVillage and self-captured images. All CNN models achieved impressive accuracy rates surpassing 95%, with DenseNet-121 particularly excelling at 99.85% accuracy. Consequently, DenseNet-121 was identified as the preferred model for incorporating into an intuitive mobile app for detecting tomato leaf diseases. The application features a user-friendly interface that is accessible in English as well as two prominent Indian languages, thereby facilitating accessibility for a broader range of users.

To optimize tomato plant productivity, an image processing-based approach was proposed by Rahman et al. [12] for the quick detection, and diagnosis of diseases of tomatoes leaf. They use the Grey Level Co-Occurrence Matrix (GLCM) approach to first extract statistical features, and then the algorithm known as Support Vector Machines to categorize the data. With 100% accuracy in recognizing healthy leaves, 95% accuracy in identifying early blight, 90% accuracy in identifying septoria leaf spot, and 85% accuracy in identifying late blight, the results are impressive. Notably, this research also sparked the creation of a smartphone app that makes disease management in tomato crops more useful and efficient.

Using deep learning techniques for the categorization and segmentation of leaf image, Shoaib et al.'s [13] goal is to detect tomato plant diseases. Plants are essential to the production of food worldwide, yet diseases result in significant losses. Manual monitoring is cumbersome and prone to error. Deep learning in machine vision reduces the risk of disease by suggesting a method based on images of tomato leaves. Supervised learning recognizes illnesses using Inception Net and semantic segmentation (U-Net, Modified U-Net). The Modified U-Net ensures high precision. With accuracy rates of 99.95% for binary classification and 99.12% for six-level classification, InceptionNet1 exceeds existing plant disease classification methods.

With improved deep learning algorithms for tomato leaf disease identification, Tarek et al. [14] conducted the mounting issues of climate change and food security. Their research, which aimed to increase productivity, concentrated on tomato crop illnesses in Egypt. They tested various pre-trained models with a focus on MobileNetV3, which had a high level of accuracy. Significantly, they assessed latency on workstations and Raspberry Pi, demonstrating the IoT possibilities at the nexus of deep learning, smart agriculture, and disease detection.

To overcome CNNs' resource constraints, Bhujel et al. [15] suggested a simple attention-based CNN for diagnosing tomato leaf disease. With the help of disease detection, this research seeks to improve agricultural security. Particularly in interclass recall and overall accuracy, which exceeds 1.1%, the lightweight CNN with an attention module greatly enhances accuracy. Averaging 99.69% detection accuracy, attention processes—and CBAM in particular—show extraordinary performance. When compared to ResNet50, these models effectively minimize the number of variables and the degree of complexity, providing an effective means of diagnosing plant diseases.

In response to COVID-19′s global effects on grain exports and food security, Zhou et al. [16] developed an original method for identifying leaf diseases of tomatoes through a restructured deep residual dense network. Grain output needs to be increased as worries about the global food supply grow. Crop diseases, however, present a substantial obstacle for farmers. Effective illness management depends on an accurate assessment of disease severity. To achieve a stunning 95% top-1 average accuracy on the Tomato test dataset, the paper develops a hybrid deep learning model that combines the benefits of dense and residual networks while minimizing training parameters. This method addresses crucial issues with food security and disease prevention while increasing crop production and productivity.

Improvement of pre-trained convolutional neural network for tomato leaf disease detection was published by Ahmad and co-authors et al. [17]. The need for accurate disease detection has a significant impact on production quantity and quality. This study explored ResNet, Inception V3, VGG-16, and VGG-19 architectures using Convolutional Neural Networks (CNN) to classify tomato leaf blight. The study evaluated the model are using datasets from the lab and field through feature extraction and parameter tuning. However, Inception V3 showed potential for correct disease classification across both datasets, although all designs performed well on lab data.

A technique for identifying and categorizing tomato plant leaf diseases using deep learning convolutional neural networks (CNNs) was put out by Salih et al. [18]. The study emphasizes the necessity for accurate disease identification in light of the significance of tomato harvests. Diseases like late blight and bacterial spot can have a big effect on tomato plants. Better productivity depends on early detection. The study uses CNNs to identify diseases based on their features and achieves a classification accuracy of 96.43%. The performance of CNN is supported by actual photographs of widespread plant diseases.

Batool et al. [19] advanced classification system was developed in this work to recognize and group diseases that damage tomato leaves. For the study, the datasets were split into nine different files, each representing a common tomato leaf disease. 450 images formed the training dataset, where visual features were retrieved using several models. Then, these features were combined with k-nearest neighbors (KNN) method to accurately classify the diseases. Among the numerous models tested, the AlexNet model emerged as the most accurate classifier. Notably, the classification accuracy of the AlexNet model for detecting tomato leaf blight was 76.1% compared to other models.

Guerrero-Ibañez et al. [20] addressed the challenge of identifying tomato leaf diseases in Mexico's agriculture using deep learning. The authors emphasize the significance of developing a deep image analysis approach to accurately classify various types of tomato leaf diseases, given the similarities in leaf deterioration across different pathogens. The CNN architecture comprises four modules: dataset creation, model creation, data distribution, and processing for optimization and performance verification. Their proposed CNN-based model leverages a public dataset and additional field photographs, achieving over 99% accuracy in disease classification. By employing generative adversarial networks (GANs) to prevent overfitting, the model accurately classifies nine disease types with high precision and recall. This research presents a promising approach to automating disease identification, offering potential benefits for crop yield and sustainability.

The LMBRNet architecture, proposed by M. Li et al. [21] introduce LMBRNet, a novel architecture for tomato disease image identification, notable for its innovative design elements enhancing accuracy. Leveraging a comprehensive grouped differentiated residual (CGDR) structure and multiple residual connections, LMBRNet captures diverse features and ensures robust information transmission. Through visual enhancement techniques and deep separable convolution, the network optimizes structure, reducing parameter count while enhancing accuracy. With an identification accuracy of 99.7% on an 8000-image dataset, LMBRNet surpasses ResNet50 and GoogleNet with fewer parameters. It also outperforms advanced models like ResMLP12 and MobileNetV3, indicating potential for broad agricultural application.

Our approach excels in comprehensiveness by curating a diverse dataset, fine-tuning state-of-the-art models like EfficientNet-B0, prioritizing model interpretability through Explainable AI techniques, and deploying user-friendly web and mobile applications for accessible diagnostic tools.

## Method details

**Fig. 1:**
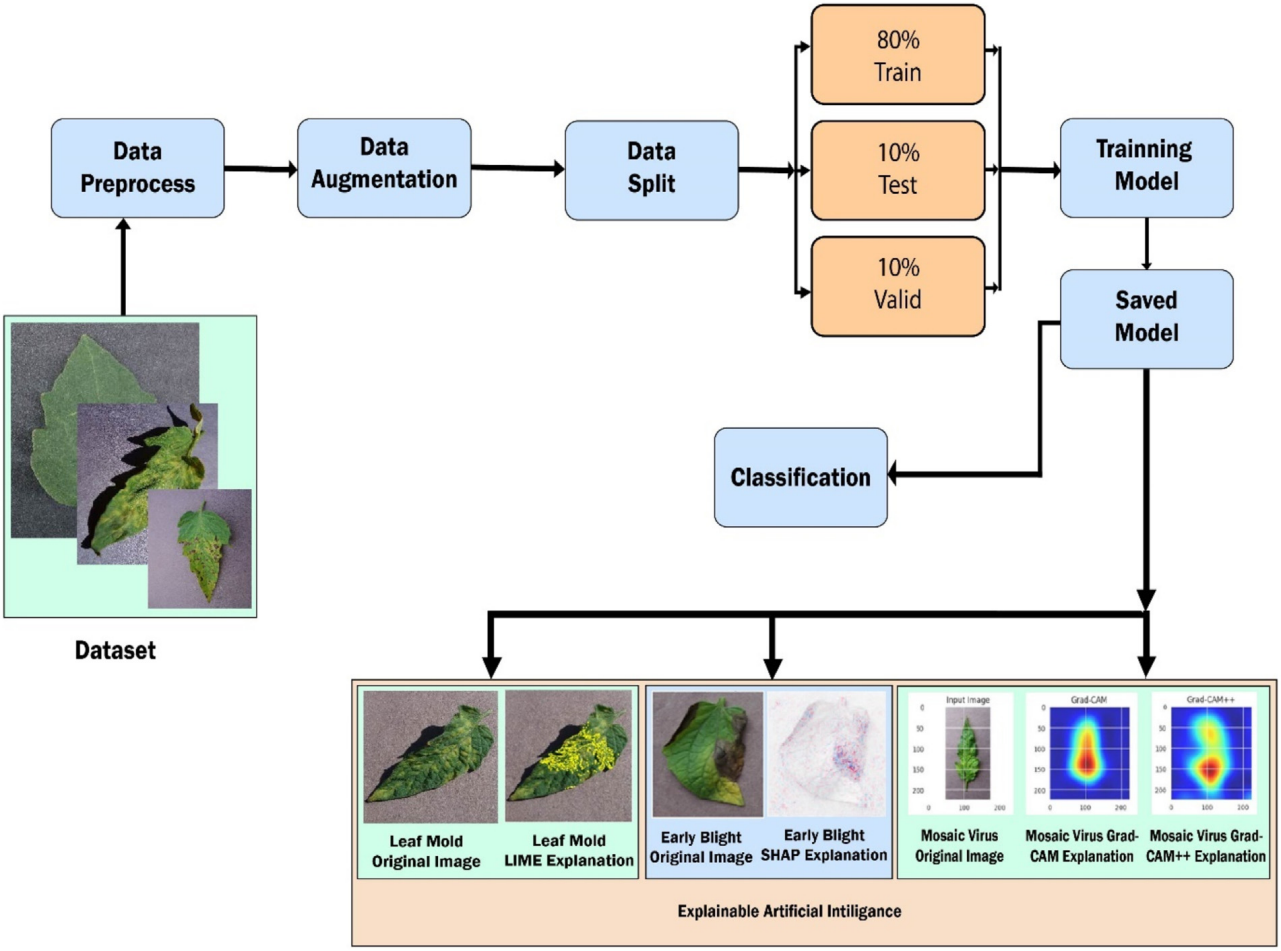
Proposed flowchart of the methodology.

A multidimensional approach that we embraced synergistically combined advanced deep learning techniques with pragmatic applications in the field of agriculture, particularly focusing on the detection and classification of tomato leaf diseases. Our methodology commenced with the meticulous curation of an extensive dataset sourced from Kaggle. This dataset, featuring a wide array of images representing eleven distinct tomato leaf conditions, formed the backbone of our study. We standardized the images to uniform dimensions, converted them into a JPEG format for uniformity and ease of processing, and implemented a variety of data augmentation techniques. These techniques, including resizing, cropping, horizontal flipping, and rotation, were instrumental in enriching the dataset's diversity, thus ensuring that our models could generalize effectively from the training data to real field conditions. At the heart of our model development process was the EfficientNet-B0. This model was meticulously chosen for its renowned balance between computational efficiency and robust performance. Alongside the EfficientNet-B0, we also experimented with other pre-trained models, including VGG19 and MobileNet, both without and with augmentation. These models underwent specific fine-tuning, particularly in layers crucial for the detection and classification of various tomato leaf diseases. The training process was optimized using the Adam optimizer, chosen for its adaptive learning rate capabilities, marking a significant enhancement over traditional methods like stochastic gradient descent. A pivotal component of our approach was the incorporation of transfer learning techniques and the integration of Explainable Artificial Intelligence (X-AI) practices. Techniques such as Grad-CAM and Grad-CAM++ were employed to bolster the interpretability of our models, allowing for a deeper understanding of the decision-making processes within the neural networks. Additionally, we utilized LIME and SHAP for further model interpretability. The culmination of our approach was the deployment of our sophisticated model into a user-friendly web-based system and Android application, providing accessible diagnostic tools for end-users in agriculture. Designed with an end-to-end (E2E) approach, our integrated system serves the dual purpose of diagnosing tomato leaf diseases and suggesting remedies, offering comprehensive agricultural aid. Fig. 1 summarizes the key aspects of our methodology, including LIME and SHAP integration for enhanced interpretability.

### Data collection and dataset description

**Fig. 2:**
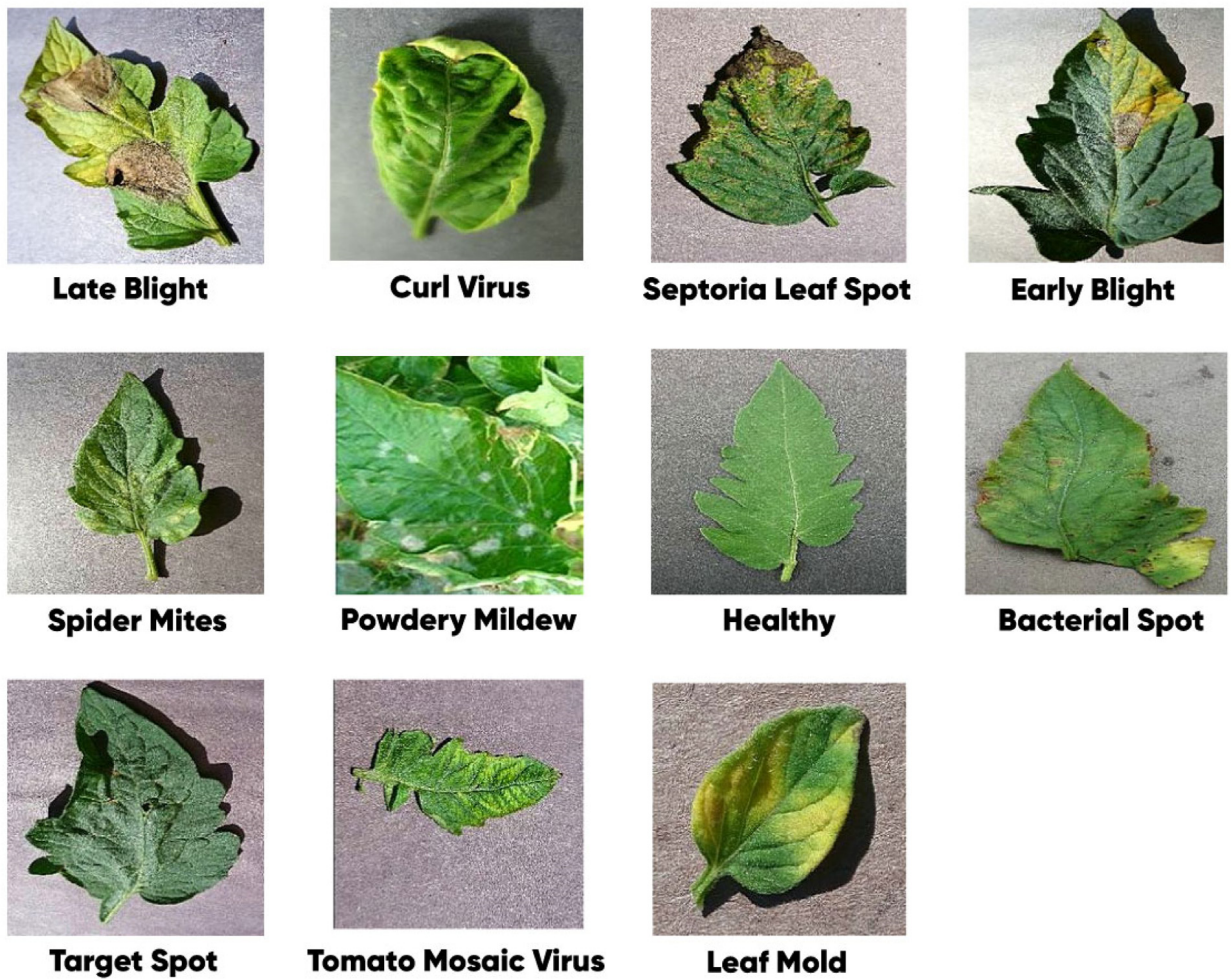
Random sample images for each tomato leaf class.

Images of tomato leaf diseases dataset were collected from a publicly available Kaggle [22]. This dataset's main goal is to make it possible to conduct in-depth studies and analyses of tomato leaf health while also making it easier to create reliable classification algorithms for disease detection. Tomato plant diseases come in all shapes and sizes. This dataset, which includes various health and disease issues that impact tomato plants, is divided into 11 different classifications. Aside from healthy tomato leaves, the dataset also contains examples of leaves with various diseases, including “Bacterial Spot,” “Early Blight,” “Late Blight,” “Leaf Mold,” “Powdery Mildew,” “Septoria Leaf Spot,” “Spider Mites Two-Spotted Spider Mite,” “Target Spot,” “Tomato Mosaic Virus,” and “Tomato Yellow Leaf Curl Virus.”. Fig. 2 illustrates the sample images of tomato leaves.

Table 1 illustrates that the dataset encompasses a total of 32,532 sample images. The training dataset is composed of 26,350 images, constituting 80% of the entire dataset. The test dataset includes 3254 images, representing 10% of the total dataset, while the validation dataset comprises 2928 images, making up the remaining 10% of the overall dataset.

**Table 1:** The dataset description for tomato leaf disease.

Class ID	Class Name	Number of Data	Training	Testing	Validating	Leaf Symptoms
Class 0	Late Blight	3905	3154	399	352	Leaf lesions, Phytophthora infestans, Brown spots, Fungal infection.
Class 1	Tomato Yellow Leaf Curl Virus	2534	2066	238	230	Yellowing leaves, Leaf curling, Stunted growth, Mottled discoloration.
Class 2	Separia Leaf Spot	3628	2954	360	314	Brown spots, Circular lesions, Fungal infection, Leaf discoloration.
Class 3	Early blight	3098	2512	323	263	Dark concentric rings, Brown lesions, Leaf yellowing, Fungal infection.
Class 4	Spider Mites TwoSpotted Spider Mite	2182	1760	220	202	Webbing, Yellow stippling, Leaf damage, Pest infestation.
Class 5	Powdery_Mildew	1256	1039	112	105	White fungal growth, powdery residue, leaf discoloration, reduced plant vigor.
Class 6	Healthy	3857	3126	391	340	green, smooth, and turgid, with strong veins, a uniform shape, and no visible signs of pests, diseases, or stress.
Class 7	Bacterial Spot	3558	2876	363	319	Circular lesions, water-soaked spots, leaf discoloration, bacterial infection.
Class 8	Target Spot	2284	1840	238	206	Concentric rings, dark circular lesions, leaf browning, fungal infection.
Class 9	Tomato Mosaic Virus	2737	2195	266	276	Mottled leaf patterns, mosaic-like discoloration, stunted growth, viral infection.
Class 10	Leaf Mold	3493	2828	244	321	Fuzzy white or gray mold, yellowing leaves, reduced vigor, fungal infection.
		Total: 32,532	Total: 26,350	Total: 3254	Total: 2928	

### Data preprocessing

The development of a precise tomato leaf disease classification model necessitates meticulous data preprocessing to effectively prepare raw image data for training and evaluation. Initially, a diverse dataset containing healthy and disease-affected tomato leaf images is collected and organized into subdirectories, each corresponding to specific disease labels, ensuring proper association of ground truth information during training. To facilitate the neural network's understanding, a label mapping process is conducted, creating a dictionary linking disease labels with unique integer IDs. Subsequently, the dataset is loaded into memory, recording file paths and integer-encoded labels for each image. Data augmentation techniques [23] like random resizing, cropping, horizontal flipping, color jittering, and random rotation are applied to enhance model generalization. Post-augmentation, images are resized to a uniform size of 240×240 pixels and standardized using mean and standard deviation values computed from the entire dataset. Subsequently, the dataset is split into training, validation, and testing sets, each serving specific purposes in the model's construction. Finally, the preprocessed dataset is structured into batches using Data Loader instances, optimizing memory efficiency and allowing parallel processing. Through these comprehensive preprocessing steps, the raw image data is transformed into a well-structured, augmented, and normalized dataset, providing the foundation for training and evaluating the fine-tuned EfficientNet-B0 model in the effective classification of tomato leaf diseases. Table 2 presents the hyperparameter and its associated value applied in this investigation.

**Table 2:** Hyperparameter for image augmentation.

Techniques	Parameter
Resize	240×240
Scale	0.8, 1.0 or 80%, 100%
Horizontal Flip	True
Rotation	15°
Brightness	0.2 or 20%
Contrast	0.2 or 20%
Saturation	0.2 or 20%
Hue	0.1 or 10%

### Proposed Model

The proposed model for tomato leaf disease classification consists of two main components: the feature extraction module based on EfficientNetB0 and the Squeeze-and-Excitation (SE) module. The EfficientNetB0 backbone is used to extract features at several scales, capturing important information from different levels of abstraction in the network. Following that, the SE module is incorporated to augment the representational capacity of the retrieved characteristics. The SE module comprises global average pooling, fully connected layers, and a channel-wise recalibration process that accentuates useful features while diminishing less pertinent ones. The recalibrated features obtained from different scales are combined, and the final classification is carried out using a fully linked layer with a softmax activation function. This section provides a comprehensive explanation of each component in the model (Table 3).

**Table 3:** For the proposed model, a training parameter after ablation study.

Parameter	Description
Optimization algorithms	Adam Optimizer
Number of epochs	20
Batch Size	64
Loss function	Categorical cross-entropy
Dropout rate	0.5 or 50%
Activation function (Hidden layers)	Relu
Activation function (Output layer)	SoftMax

### Proposed architecture

**Fig. 3:**
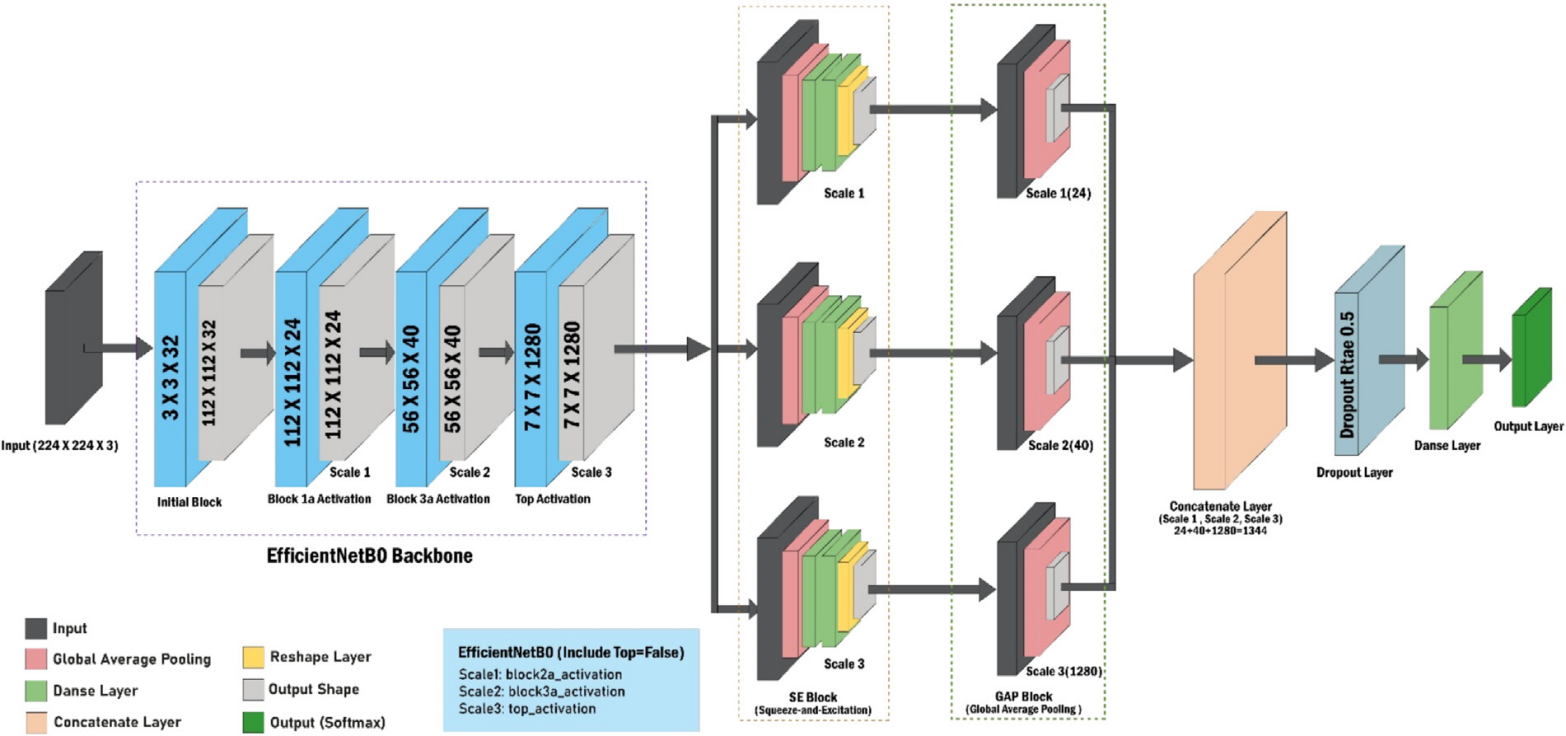
Proposed model architecture.

The input images undergo preprocessing to achieve a consistent size of 224×224 pixels, with three color channels (RGB), to match the input specifications of the EfficientNetB0 architecture (shown in Fig. 3). The backbone of the model is EfficientNetB0, with the include_top parameter set to False to exclude the default fully connected layers. This enables us to extract features from various layers of the network, thereby capturing varying levels of abstraction.

Multi-Scale Feature Extraction: In order to capture both high-level semantic information and low-level details, multi-scale features [24] are extracted from various phases of the EfficientNetB0. Basic textural details are captured by extracting early features from block2a_activation. In order to obtain more comprehensive structural information, intermediate features are extracted from block3a_activation. The top_activation yields deep semantic features, which are the higher-order features that are crucial for precise classification. This equation represents the extraction of early features, capturing basic textural details from the layer block2a_activation.

$$F_{early}=f_{EffNet}^{block2a}\left(X_{input}\right)$$

This equation captures more comprehensive structural information from the layer block3a_activation:

$$F_{intermediate}=f_{EffNet}^{block3a}\left(X_{input}\right)$$

This equation represents the extraction of deep semantic features, crucial for precise classification, from the layer top_activation.

$$F_{deep}=f_{EffNet}^{topactivation}\left(X_{input}\right)$$

Squeeze-and-Excitation (SE) Blocks: In order to improve the model's representational capacity, SE blocks [25] were incorporated to enhance the extracted features. The global average pooling is applied to each feature map using the following equation:

$$z_{c}=\frac{1}{H\times W}\sum_{i=1}^{H}\sum_{j=1}^{W}F_{c}\left(i,j\right)$$

Where:

H and W are the height and width of the feature map.

$F_{c}$ is the feature map for channel c.

$z_{c}$ is the pooled value for channel c.

The SE block utilizes a method of channel-wise feature recalibration. It starts by applying global average pooling to compress the spatial dimensions. Then, it employs two fully connected layers to capture the interdependencies between channels. The recalibrated features are multiplied with the original input to emphasize informative features and diminish less important ones.SE blocks were implemented on all the extracted feature maps, starting from the early, intermediate, and final layers, before moving on to the next stage. The SE block recalibrates the channel features using fully connected layers, as represented by the following equation:

$$s=\sigma \left(W_{2}\cdot ReLU\left(W_{1}\cdot z\right)\right)$$

Where:

z is the vector of global pooled features.

$W_{1}$ and $W_{2}$ are the weights of the fully connected layers.

σ is the sigmoid function.

The recalibrated feature map is obtained by:

$$F_{SE}=F⊙s$$

Where ⊙ denotes channel-wise multiplication.

Global Average Pooling and Feature Fusion: The recalibrated feature maps from each SE block underwent Global Average Pooling [26] to decrease the spatial dimensions and obtain a concise feature vector for each scale. The pooled feature vectors were combined to create a comprehensive feature vector that incorporates information from various scales, enhancing its reliability for classification purposes. The global average pooling is applied to each feature map using the following equation:

$$z_{c}=\frac{1}{H\times W}\sum_{i=1}^{H}\sum_{j=1}^{W}F_{c}\left(i,j\right)$$

Here, H and W are the height and width of the feature map, $F_{c}$ is the feature map for channel c, and $z_{c}$ is the pooled value for channel c.

The feature vectors from different scales are concatenated to form a comprehensive feature vector using the following equation:

$$v_{fused}=\left[v_{early},v_{intermediate},v_{deep}\right]$$

Where,

$v_{early}$ represents the feature vector from the early layer.

$v_{intermediate}$ represents the feature vector from the intermediate layer.

$v_{deep}$ represents the feature vector from the deep layer.

The operation [·;·;·] denotes the concatenation of these vectors along a specific axis, typically the feature dimension, resulting in a single, fused feature vector $v_{fused}$ that incorporates information from all scales.

Regularization and Classification: In order to mitigate overfitting and enhance generalization, the fused feature vector was subjected to a dropout layer with a dropout rate of 0.5. The probability distribution over the 11 classes was outputted by the final classification, which was performed using a Dense layer and a softmax activation function. Dropout is applied to the fused feature vector using the following equation:

$$v_{drop}=Dropout\left(v_{fused\;},p=0.5\right)$$

Here, p represents the dropout rate, which is set to 0.5 to prevent overfitting.

The final classification is performed using a dense layer with softmax activation, represented by the following equation:

$$\hat{y}=Softmax\left(W_{dense}\cdot v_{drop}+b_{dense}\right)$$

In this equation, $W_{dense}$ and $b_{dense}$ are the weights and biases of the dense layer, and $\hat{y}$ is the output probability distribution over the 11 classes.

The Adam optimizer [27] was employed to construct the final model, which accommodates the learning rate during training and employs categorical crossentropy as the loss function, making it suitable for multi-class classification tasks. The accuracy of the model was assessed by determining the percentage of samples that were correctly classified. The equation for categorical cross-entropy as the loss function is given by:

$$L=-\sum_{c=1}^{C}y_{c}log\left({\hat{y}}_{c}\right)$$

Where,

L represents the categorical cross-entropy loss.

C is the total number of classes.

$y_{c}$ is a binary indicator (0 or 1) that denotes whether the correct class label is c for a given sample.

${\hat{y}}_{c}$ is the predicted probability for class c, as output by the softmax activation function.

The summation $\sum_{c=1}^{C}\;$runs over all classes.

The Adam optimizer updates the model parameters using the following equation:

$$\theta_{t+1}=\theta_{t}-\eta \frac{\hat{m}}{\sqrt{{\hat{v}}_{i}}+\varepsilon }$$

Where $\theta_{t}$ are the model parameters at time step t,η is the learning rate, ${\hat{m}}_{t}$ and ${\hat{v}}_{t}$ are the estimates of the first and second moments of the gradients, and ϵ is a small constant to prevent division by zero.

The architecture of the model consists of a pre-trained base from EfficientNetB0, SE blocks for feature recalibration, and a fully connected classifier. This combination results in a strong and reliable solution for classifying diseases in tomato leaves. Utilizing multi-scale feature extraction and feature fusion improves the model's capacity to generalize across multiple disease categories, making it highly suitable for practical applications in agricultural environments.

#### Transfer learning

Transfer learning [28] is a technique that allows knowledge gained from training one model on one task to be used in another activity that is unrelated but yet similar. For applications like image recognition, transfer learning in the context of neural networks entails using pre-trained models that have already been trained on big datasets. Transfer learning's main goal is to use the pre-trained model's learned features and representations to refine them on a new, smaller dataset or for a different task . When we have little data for the new assignment, this can greatly speed up the training process and enhance the model's performance. When we don't have enough data to create a complicated model from scratch or we want to take advantage of the knowledge that models that have been trained on massive datasets have captured, transfer learning is especially helpful. It's an effective strategy for hastening model development and improving performance with constrained resources.

#### VGG19

The VGG19 [29] convolutional neural network, developed by Oxford's Visual Geometry Group, is well-known for image classification tasks due to its 19 layers, which include convolutional and fully connected ones. It is well-known for its uniform architecture, which consists mostly of 3×3 convolutional filters with a total of 16 layers, followed by rectified linear unit (ReLU) activation functions. These layers are interspersed by 2×2 max-pooling filters, which reduce spatial dimensions. Despite its high computational demand and memory constraints, VGG19 culminates in a softmax output layer for classification with three fully linked layers of 4096 neurons each with ReLU activations.

#### MobileNetV2

MobileNetV2 [30] is a convolutional neural network architecture specifically designed for mobile and embedded devices with limited computational resources. Developed by Google, MobileNet aims to achieve high efficiency while maintaining competitive accuracy in image classification tasks. It introduces depthwise separable convolutions, which decompose standard convolutions into separate depthwise and pointwise convolutions. This design significantly reduces the computational cost and number of parameters while preserving representational capacity. MobileNet architectures come in various sizes, denoted by a multiplier parameter that controls the number of filters in each layer. The smaller versions of MobileNet offer faster inference speeds with a trade-off in accuracy, making them suitable for real-time applications on resource-constrained devices. MobileNet has been widely adopted in various computer vision tasks, including object detection, semantic segmentation, and facial recognition, demonstrating its versatility and effectiveness across different domains and applications*.*

### Explainable artificial intelligence (XAI)

Explainable artificial intelligence, known as XAI, is an area of expertise that pertains to the advancement of AI systems with the ability to furnish explicit and comprehensible reasoning for decisions and predictions. Its fundamental objective is to guarantee accountability and transparency in AI systems, enabling users to have faith in and understand their decisions. To provide both global and regional explanations for the predictions proposed model, we utilized two well-known explainable AI approaches, LIME and SHAP. SHAP offers an integrated framework for evaluating the significance of features. While LIME uses a local linear technique to generate a model that can be interpreted. Furthermore, we utilized Grad-CAM and Grad-CAM++ [31] techniques to emphasize the areas of the input image that have a significant impact on the model's predictions.\, contributing significantly to AI system interpretability and trustworthiness.

#### SHAP

SHAP (Shapley Additive exPlanations) [32] is a machine learning framework that provides a clear understanding of how each feature contributes to a model's output. This method computes Shapley values, which quantify the cumulative marginal influence of an attribute across nearly all potential feature combinations, by cooperative game theory. SHAP values offer a reliable and uniform method for assigning the model's prediction to specific features. This approach helps in comprehending the influence of each feature on predictions, promoting transparency and enabling model interpretation. It has been widely used in different fields, adding to the increasing focus on explainability in machine learning.

#### LIME

LIME [33] is a popular model-independent approach to explaining individual predictions given by a black-box model. This technique is a beneficial tool in machine learning since it enables the approximation of complicated models by training local, interpretable models around individual data points. This provides useful insights into the model's behavior in individual instances. This approach improves model transparency and is especially valuable for comprehending predictions in black-box models.

### Grad CAM

Grad-CAM (Gradient-weighted Class Activation Mapping) is an interpretative method utilized in computer vision to visualize and comprehend Convolutional Neural Networks' decision-making procedures. Unlike earlier techniques focusing on examining features at the final convolutional layer, Grad-CAM operates by computing gradients directed towards the last convolutional layer, aiming to discern the significance of each feature map. This approach generates a heatmap pinpointing the crucial image regions that substantially influenced the model's classification verdict. By analyzing these gradients, Grad-CAM highlights specific areas in an image that significantly impacted the model's decision, offering visual explanations for the model's predictions. Its utilization spans diverse domains like healthcare, object recognition, and autonomous driving, playing a pivotal role in enhancing the interpretability of deep learning models. We can integrate Grad-CAM through this step implementation as outlined below:

Step 1: The derivative of the class score (denoted by 'c') concerning the activation feature maps within a convolutional layer (Compute Gradient):
  (5) $$\frac{\delta y^{c}}{\delta A^{k}}$$
Step 2: Gradient calculation: αkc = (1/N) ∑ (i, j) (∂yc/∂Aij^k)
  (6) $$a_{k}^{c}=\frac{1}{z}\sum_{i}\sum_{j}\;\frac{\delta y^{c}}{\delta A_{ij}^{k}}$$
Step 3: Global average pooling: αkc = average(αkc_ij)
  (7) $$L_{Grad-Cam}^{c}=ReLU\left(\sum_{k}a_{k}^{c}A^{k}\right)$$
Step 4: Weighted combination: Lc_Grad-CAM = ReLU(∑k αkc * Ak)
  (8) $$Grad-CAM^{cat}=ReLU^{2}\left(\alpha_{1}A^{1}+\alpha_{2}A^{2}+\alpha_{3}A^{3}\right)$$

#### Grad-CAM++

Grad-CAM++ represents an improved iteration of Grad-CAM, offering more precise and localized visual explanations for the model. It concentrates on positive gradients, producing sharper heatmaps that distinctly showcase the exact influential regions affecting model predictions. This enhancement results in superior localization accuracy, especially beneficial for scenarios involving multi-instance object detection, and offers enhanced visual representations of the models for making decision procedures. Grad-CAM++ is a valuable tool that enhances model interpretability by providing more comprehensive insight into the decision-making process of models.

### Evaluation matrix

The performance of a statistical, machine learning, or deep learning model is assessed using evaluation metrics. A variety of assessment metrics need to be used to examine the proposed model. Evaluation metrics are essential to establishing model performance. Performance evaluation metrics for classification or prediction models consist of f1-score, recall, precision, and accuracy [34].

#### Accuracy

The percentage of images out of all forecasts that were successfully predicted is known as accuracy. The accuracy is indicated by the following Eq. (9):

(9) $$Accuracy=\frac{True\;Positive+True\;Negative}{True\;Positive+True\;Negative+False\;Positive+False\;Negative}$$

The percentage of a model's predictions that were accurate relative to all other forecasts is measured by the accuracy score.

#### Recall

Through an investigation of the overall count of valid samples that are positive (True Positive + False Negative) to the number of true positive findings, the value of recall is employed in assessing the precision of positive forecasting. For calculating the recall value, use Eq. (10) as a guide:

(10) $$Recall=\frac{True\;Positive}{True\;Positive+False\;Negative}$$

#### F1-Score

Researchers use measures such as the F1-score for measuring the model's performance. The cumulative average of the accuracy of the model and recall is used to get the F1 score (in Eq. 11).

(11) $$F1-Score=2\times \frac{Recall\times Precision}{Recall\pm Precision}$$

### Method validation

The result analysis offers a comprehensive insight into the performance and results of the classification model for tomato leaf disease, developed using the distinctive architecture of EfficientNet-B0. It covers diverse elements such as classification metrics, trends in loss and accuracy, as well as visualizations, unveiling valuable insights into the model's behavior, strengths, and areas that could be enhanced.

#### Classification report

A key element of the outcome analysis is the classification report. It offers a thorough analysis of how each class in the dataset performed. The classification report offers a detailed examination of the model's ability to differentiate between various classes, utilizing metrics such as Recall, Precision, F1-score, and Support [29]. It provides insights into the model's performance on individual classes, highlighting any imbalances or challenges encountered in specific disease categories.

#### Classification matrix

When dealing with multiple classes, the confusion matrix serves as a crucial instrument for evaluating the classification models' performance [35]. The model's estimates are compared to the actual labels for each class in detail. The confusion matrix is pivotal in understanding the strengths and weaknesses of the model's classification abilities, allowing for a deeper analysis of its performance. Here we are computed and visualized a confusion matrix for the classification model using the seaborn library.

**Fig. 4:**
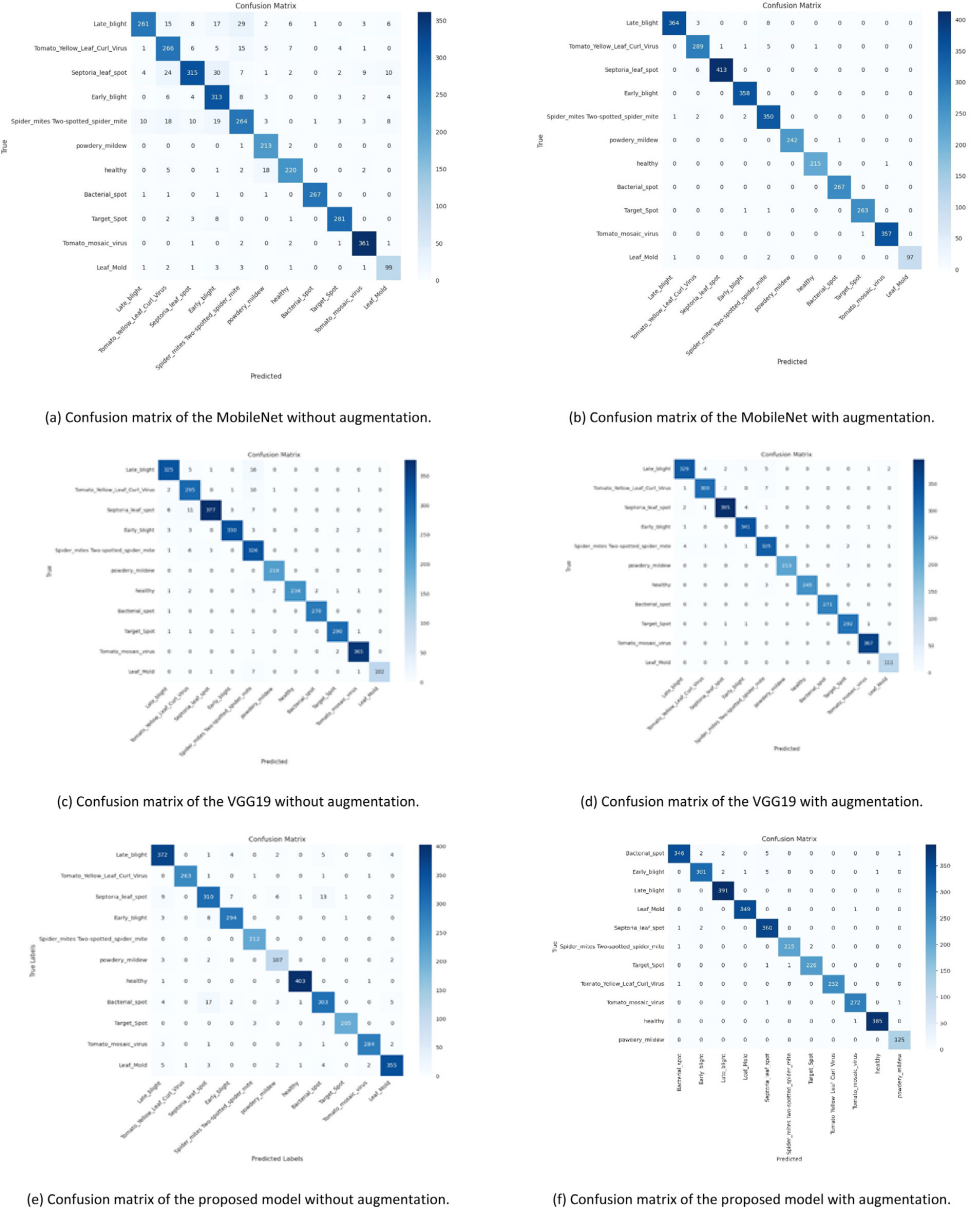
Confusion matrix of MobileNet, VGG19, EfficientNet-B0 with and without augmentation.

Fig. 4 shows that using data augmentation techniques on cutting-edge models significantly improves their performance. Without utilizing data augmentation techniques, among the models, our proposed model has performed best. However, when data augmentation is used, fine-tuned EfficientNet-B0 model's performance improves significantly, with near-perfect accuracy in predicting test samples.

#### Loss and accuracy graph

The plotted loss and accuracy trends over epochs provide a visual representation of the model's learning process. Analyzing these trends helps us understand how the model evolves during training, validation, and testing. Loss plots show how the model's error decreases over epochs, while accuracy plots show how closely the model's predictions coincide with the true labels. Discrepancies between training, validation, and testing trends may indicate overfitting or underfitting, offering insights into model performance and generalization.

**Fig. 5:**
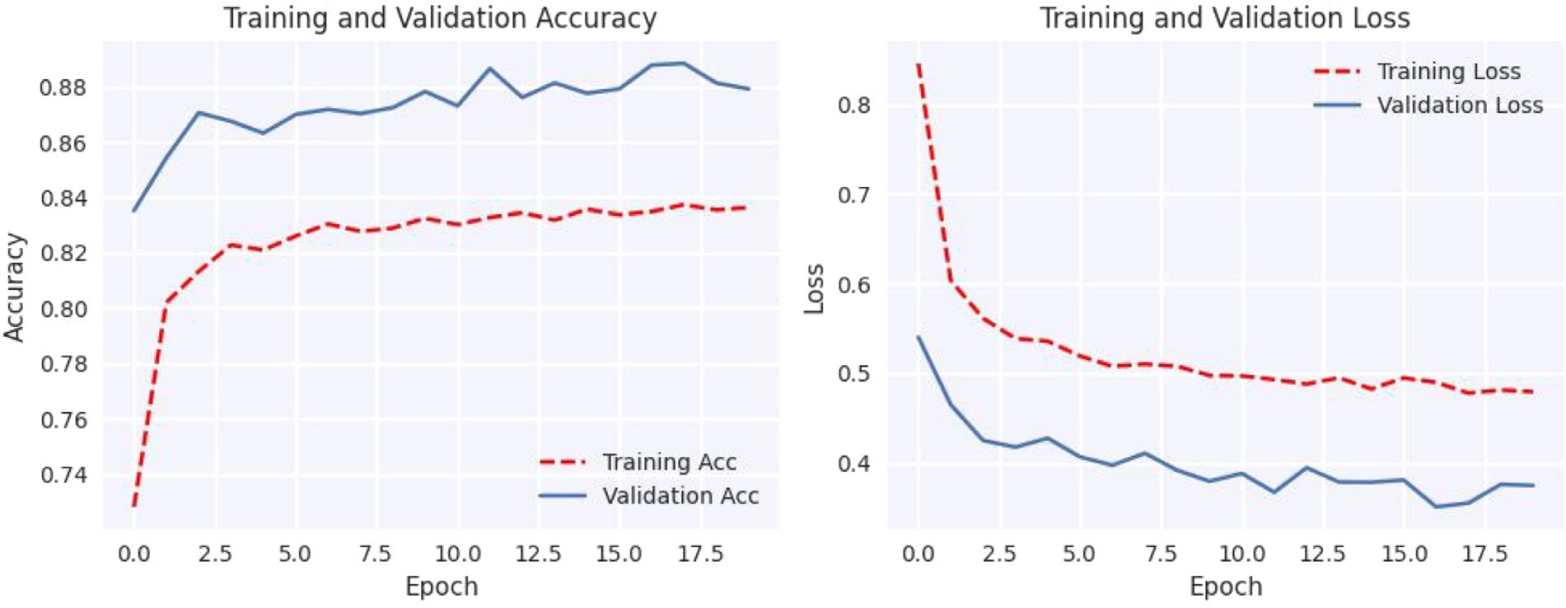
MobileNet without augmentation Training vs Validation - Loss, Accuracy Graph.

**Fig. 6:**
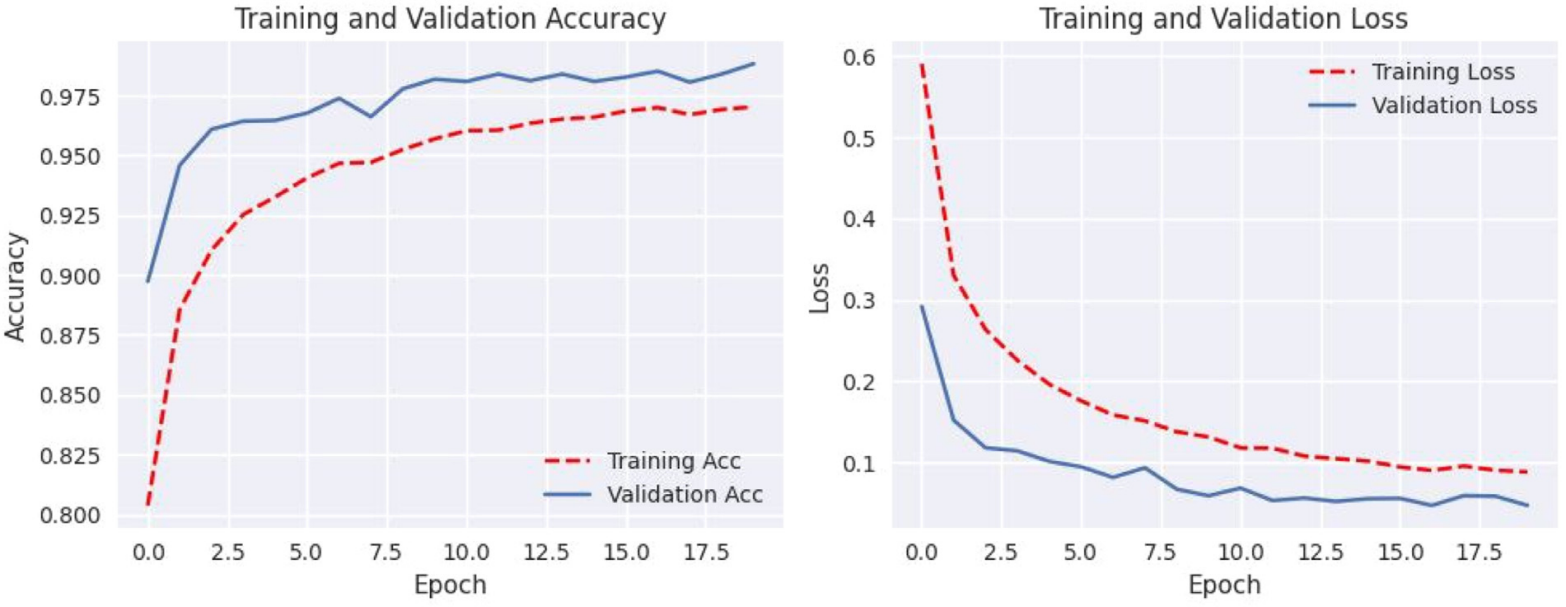
MobileNet with augmentation Training vs Validation - Loss, Accuracy Graph.

**Fig. 7:**
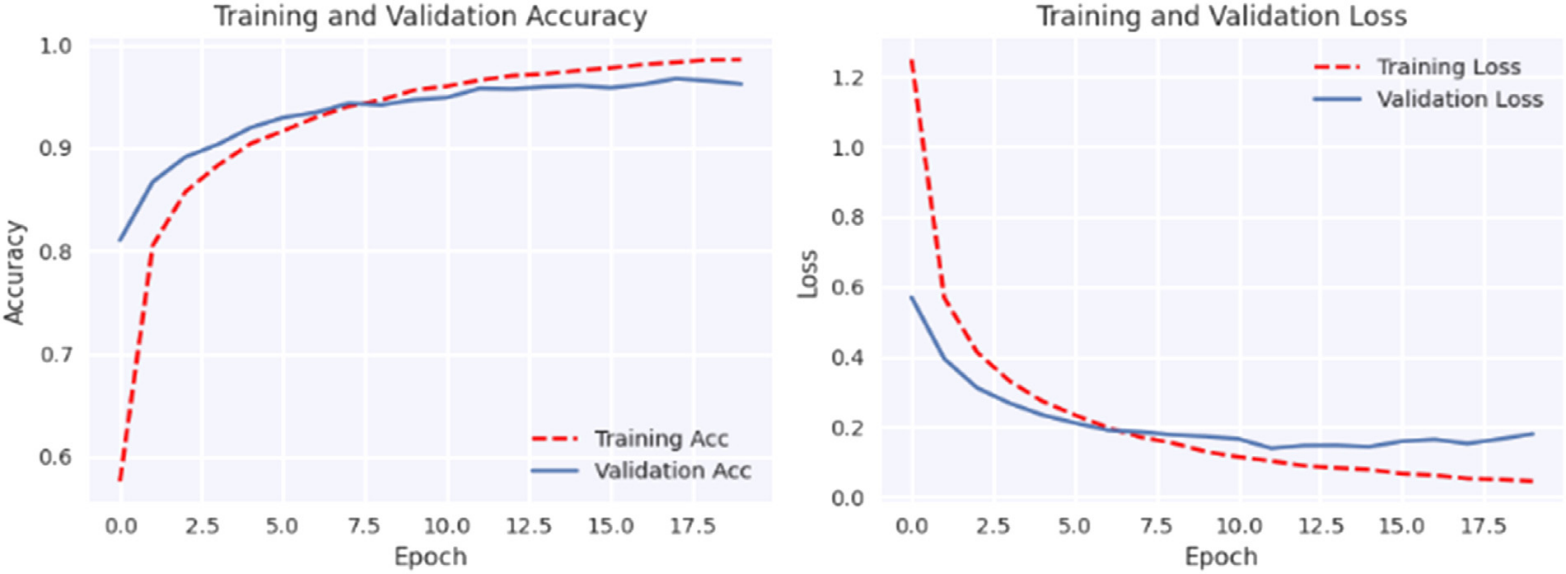
VGG19 without augmentation Training vs Validation - Loss, Accuracy Graph.

**Fig. 8:**
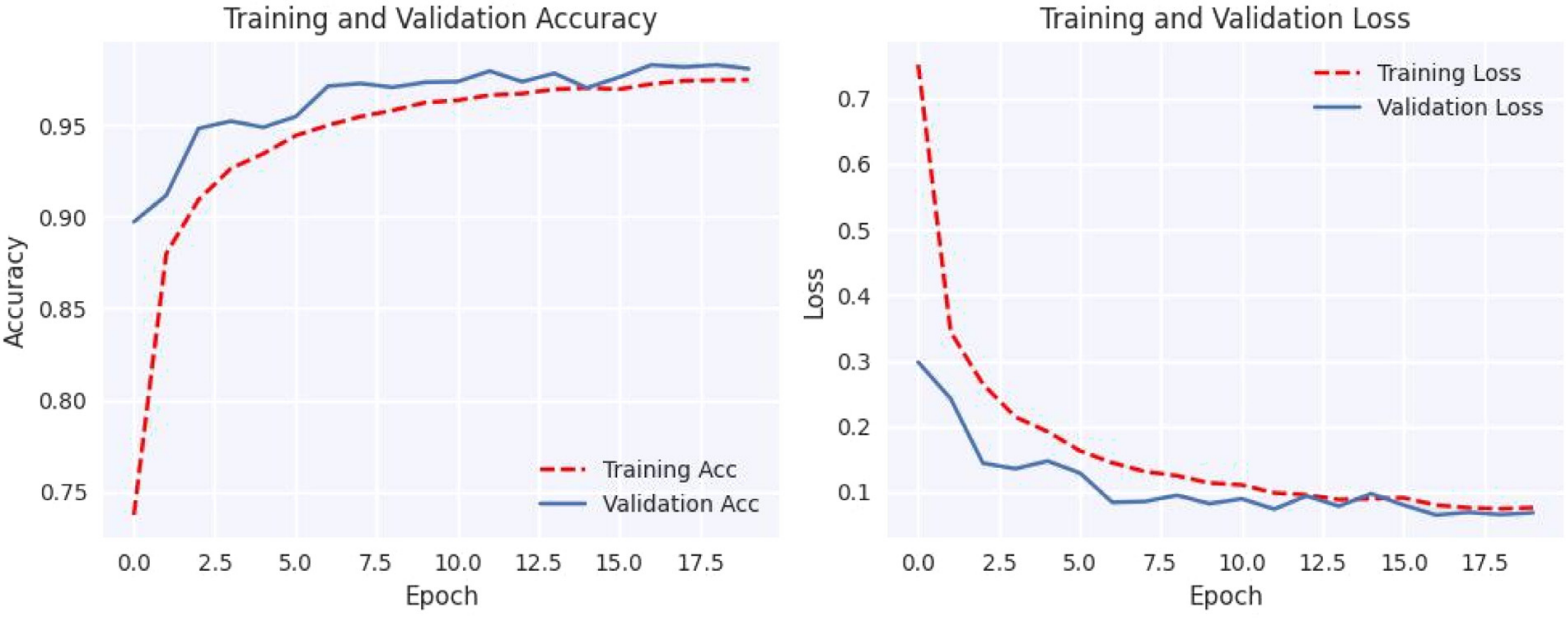
VGG19 with augmentation Training vs Validation - Loss, Accuracy Graph.

**Fig. 9:**
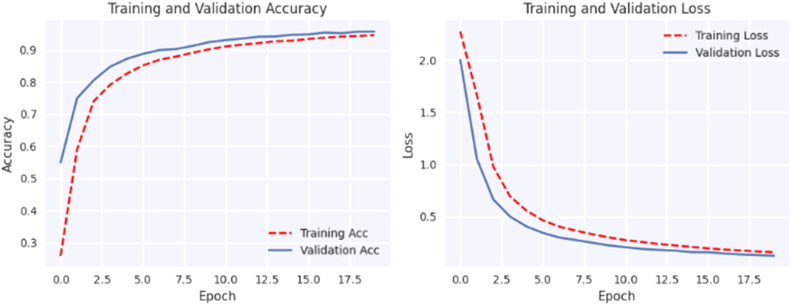
Proposed model without augmentation Training vs Validation - Loss, Accuracy Graph.

**Fig. 10:**
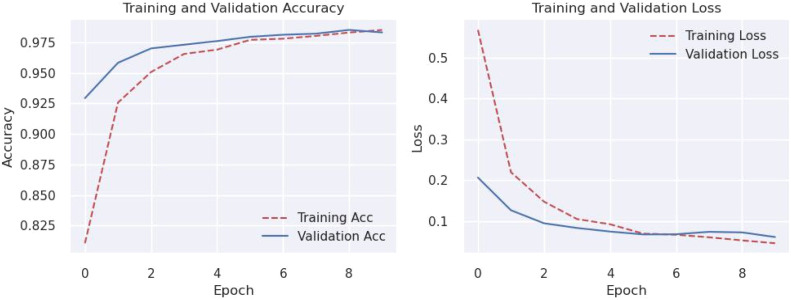
Proposed model with augmentation Training vs Validation - Loss, Accuracy Graph.

Fig. 5, Fig. 6, Fig. 7, Fig. 8, Fig. 9, Fig. 10 shows that the model trained with augmentation techniques has led to increased accuracy and confronted fewer losses than those who were not trained with augmentation. It is evident from Fig. 10 that fine-tuned Efficientnet-B0 with augmentation has attained the most desirable accuracy in comparison with the graph of accuracy for other models. Additionally, in comparison to other models, this model incorporates augmentation has seen a lower level of loss.

### Evaluation results

The model exhibits remarkable performance, with an accuracy of 99.2% on the test dataset, recall and precision both at 99%, and an F1-Score of 99%. This highlights the model's strength and dependability across different evaluation criteria. The achievement underscores the effectiveness of the tailored EfficientNet-B0 design in precisely categorizing tomato leaf diseases. Moreover, the model's ability to generalize effectively to unseen data is evident from its high accuracy, emphasizing its practical utility (refer to Table 4).

**Table 4:** Classification report.

Class Name	Precision	Recall	F1-Score	Support
Late blight	0.99	1.00	0.99	388
Tomato Yellow Leaf Curl Virus	1.00	1.00	1.00	275
Septoria leaf spot	0.97	0.99	0.98	348
Early blight	0.99	0.97	0.98	296
Spider mites Two spotted spider mite	1.00	0.99	0.99	207
Powdery mildew	0.98	1.00	0.99	121
Healthy	1.00	1.00	1.00	388
Bacterial spot	0.99	0.97	0.98	358
Target Spot	0.99	0.99	0.99	230
Tomato mosaic virus	0.99	1.00	0.99	291
Leaf Mold	1.00	1.00	1.00	352
Accuracy			0.99	3254
Macro avg	0.99	0.99	0.99	3254
Weighted avg	0.99	0.99	0.99	3254

The result analysis, combined with the attained accuracy of 99.11%, offers a thorough insight into the model's performance, its capabilities, and possible areas for enhancement. This analysis forms the basis for informed decision-making in refining the model architecture, hyperparameters, and data preprocessing strategies to enhance classification accuracy and address any observed limitations.

Table 5 presents the training, validation, and loss accuracy of the models along with test accuracy. Augmentation has led to a significant enhancement in the performance of the model. Models that were augmented achieved higher test accuracy compared to models that were not augmented. Among the models assessed, the proposed model, when incorporating augmentation methods, demonstrated the highest accuracy rate of 99.11%, striking a harmonious balance between accuracy and computational efficiency. This underscores the efficacy of data augmentation in enhancing model effectiveness.

**Table 5:** Accuracy and loss metrics for training and validation of models.

Model name	Accuracy	Training accuracy	Validation accuracy	Training loss	Validation loss
MobileNet model (without Augmentation)	87.44%	0.8363	0.8792	0.4794	0.3749
MobileNet model (with Augmentation)	97.80%	0.9703	0.9883	0.0888	0.0474
VGG19 model (without Augmentation)	95.50%	0.9860	0.9622	0.0449	0.1803
VGG19 model (with Augmentation)	97.01%	0.9755	0.9803	0.0741	0.0767
Proposed model (without Augmentation)	95.20%	0.9471	0.9552	0.1583	0.1171
Proposed model (with Augmentation)	99.11%	0.9997	0.9904	0.0487	0.0523

A detailed look at the class-wise assessment metrics for ResNet50, VGG-19, and the proposed model is presented in Table 6. Notably, VGG-19 exhibits higher recall values when augmentation techniques are applied than when they are not. Augmentation approaches significantly enhanced the performance of all models relative to models trained without proper augmentation. The proposed model with augmentation approaches outperforms other models and has the most promising evaluation metric values. This highlights the importance of effective data augmentation in achieving superior model performance.

**Table 6:** Recall, F1-score, and precision for each class for various algorithms.

Model Name	Name of Class	Result without augmentation			Result with augmentation		
		Precision	Recall	F1-Score	Precision	Recall	F1-Score
	Class 0	0.95	0.79	0.86	0.99	0.96	0.97
	Class 1	0.79	0.84	0.82	0.97	0.95	0.96
	Class 2	0.89	0.75	0.81	0.98	0.98	0.98
	Class 3	0.78	0.91	0.84	0.98	0.99	0.98
	Class 4	0.81	0.82	0.81	0.93	0.99	0.96
MobileNet	Class 5	0.89	0.98	0.93	0.99	0.99	0.99
	Class 6	0.93	0.90	0.91	1.00	0.99	0.99
	Class 7	0.98	0.95	0.97	1.00	1.00	1.00
	Class 8	0.95	0.97	0.96	1.00	1.00	1.00
	Class 9	0.93	0.98	0.95	0.99	1.00	1.00
	Class 10	0.86	0.92	0.89	1.00	0.96	0.98
	Class 0	0.95	0.93	0.94	0.99	0.94	0.96
	Class 1	0.92	0.96	0.94	0.97	0.97	0.97
	Class 2	0.98	0.94	0.96	0.97	0.99	0.98
	Class 3	0.96	0.96	0.96	0.98	1.00	0.99
	Class 4	0.90	0.98	0.94	0.98	0.97	0.98
VGG19	Class 5	0.99	0.99	0.99	0.99	0.99	0.99
	Class 6	0.99	0.96	0.98	0.99	0.98	0.98
	Class 7	1.00	0.98	0.99	1.00	1.00	1.00
	Class 8	1.00	0.98	0.99	0.98	0.98	0.98
	Class 9	1.00	0.99	1.00	0.99	1.00	1.00
	Class 10	0.96	0.98	0.97	0.96	1.00	0.98
	Class 0	0.95	0.97	0.96	0.99	1.00	0.99
	Class 1	0.99	0.99	0.99	1.00	1.00	1.00
	Class 2	0.91	0.89	0.90	0.97	0.99	0.98
	Class 3	0.95	0.95	0.95	0.99	0.97	0.98
	Class 4	0.99	0.98	0.98	1.00	0.99	0.99
Proposed model	Class 5	0.87	0.96	0.91	0.98	1.00	0.99
	Class 6	0.98	1.00	0.99	1.00	1.00	1.00
	Class 7	0.94	0.91	0.92	0.99	0.97	0.98
	Class 8	0.98	0.97	0.97	0.99	0.99	0.99
	Class 9	0.98	0.97	0.97	0.99	1.00	0.99
	Class 10	0.97	0.96	0.97	1.00	1.00	1.00

#### ROC-AUC curve

**Fig. 11:**
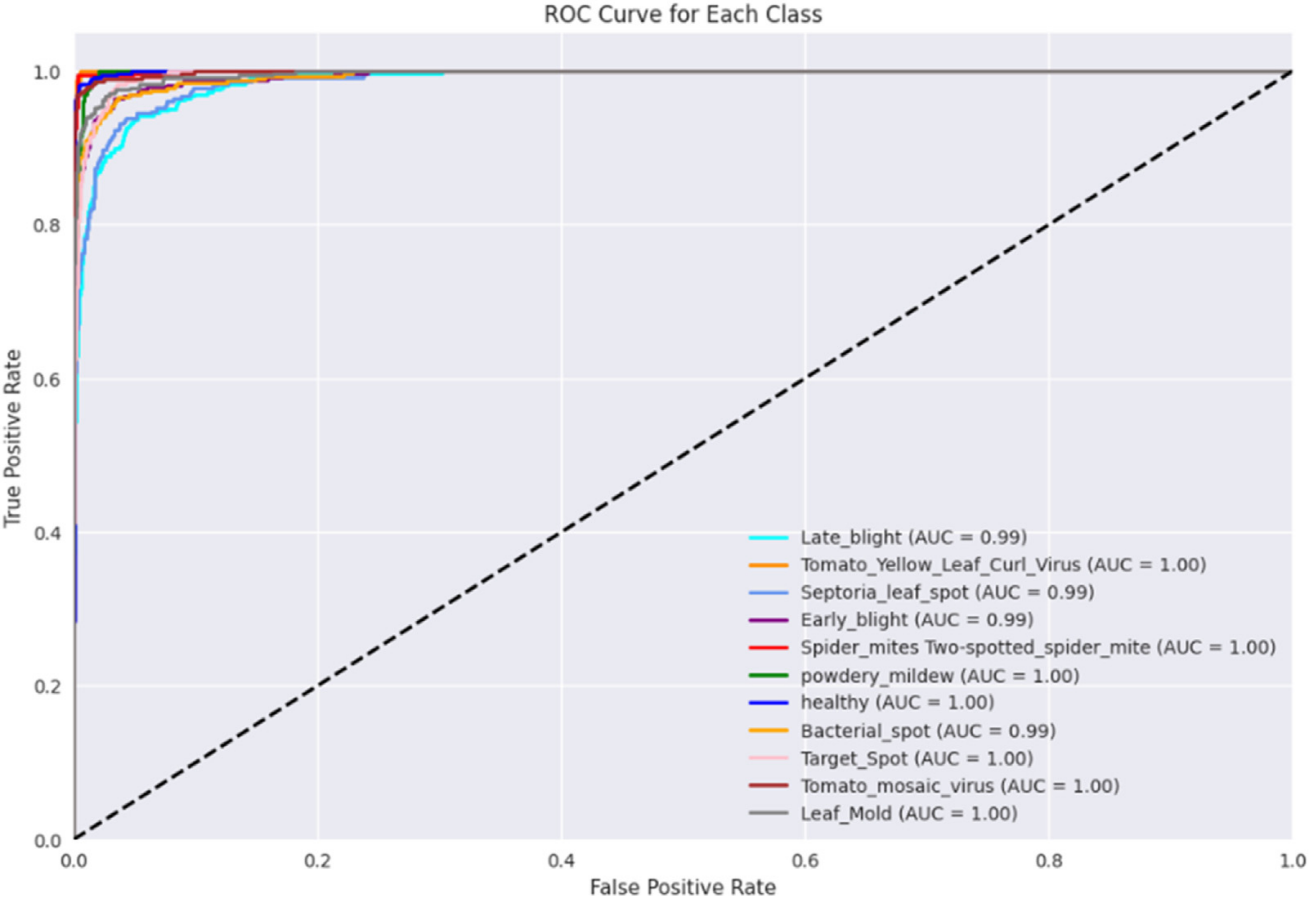
Class-wise Roc Curve output from the proposed model.

Evaluation of a classification model can be accomplished through the utilization of an ROC curve by displaying the trade-off between the true positive (TP) rate and the false positive (FP) rate. It evaluates the model's efficacy across different levels of classification [36]. The ROC curve analysis, as illustrated in Fig. 11, offers a detailed insight into the performance of our proposed method across different classes of tomato leaf diseases. Each curve represents the TP Rate contrasted with the FP Rate for a specific disease category. Notably, the proposed approach exhibits exceptional performance across all classes, as evidenced by the high AUC (Area Under the Curve) values. For instance, the AUC values for classes like Tomato Yellow Leaf Curl Virus, Spider Mites Two-Spotted Spider Mite, Powdery Mildew, and Leaf Mold are all perfect (1.00), indicating near-perfect discrimination between positive and negative instances for these diseases. Additionally, diseases like Late Blight, Septoria Leaf Spot, Early Blight, Bacterial Spot, Target Spot, and Tomato Mosaic Virus also demonstrate outstanding discrimination capability, with AUC values close to 1.00. This comprehensive analysis underscores the effectiveness and reliability of our proposed approach in accurately identifying various tomato leaf diseases, highlighting its potential for practical deployment in agricultural settings.

**Evaluation of Model Overfitting Using Cross-Validation:** To assess the potential overfitting of the tomato leaf disease detection model, stratified cross-validation [37] was applied with different fold configurations, specifically 5-fold and 10-fold. The aim was to observe the variation in training and validation accuracies across different splits and identify any significant discrepancies that could indicate overfitting. The findings of the cross-validation approaches for 5-fold, and 10-fold are shown in Table 7, Table 8.

**Table 7:** The 5-fold cross-validation results.

Accuracy	F-1	F-2	F-3	F-4	F-5	Average
**Training**	0.9894	0.9950	0.9969	0.9925	0.9967	0.9941
**Validation**	0.9677	0.9766	0.9883	0.9946	0.9902	0.9835

**Table 8:** The 10-fold cross-validation results.

Accuracy	F-1	F-2	F-3	F-4	F-5	F-6	F-7	F-8	F-9	F-10	Average
**Training**	0.9901	0.9909	0.9945	0.9952	0.9963	0.9931	0.9945	0.9935	0.9949	0.9974	0.9936
**Validation**	0.9658	0.9831	0.9885	0.9896	0.9912	0.9908	0.9969	0.9950	0.9915	0.9958	0.9888

For the 5-fold cross-validation, the average training accuracy was approximately 0.9941, and the average validation accuracy was 0.9835. The gap between the training and validation accuracies remained small, with a difference of about 0.0106 on average, indicating good generalization of the model to unseen data. On the other hand, the average training accuracy was 0.9936, while the average validation accuracy was 0.9888 for the 10-fold cross validation. The gap between training and validation accuracies was slightly smaller than in the 5-fold case, with an average difference of 0.0048. In both cases (5-fold and 10-fold), validation accuracies for both folds above 98%. Moreover, as the number of folds increased, the validation accuracy became more consistent, indicating that the ability of the model to generalize improved with more data splits. This shows that our proposed model is robust and generalizes effectively to unseen data, with minimal overfitting observed and also maintains consistent performance across different cross-validation configurations.

#### Visualization results using LIME and SHAP

**Fig. 12:**
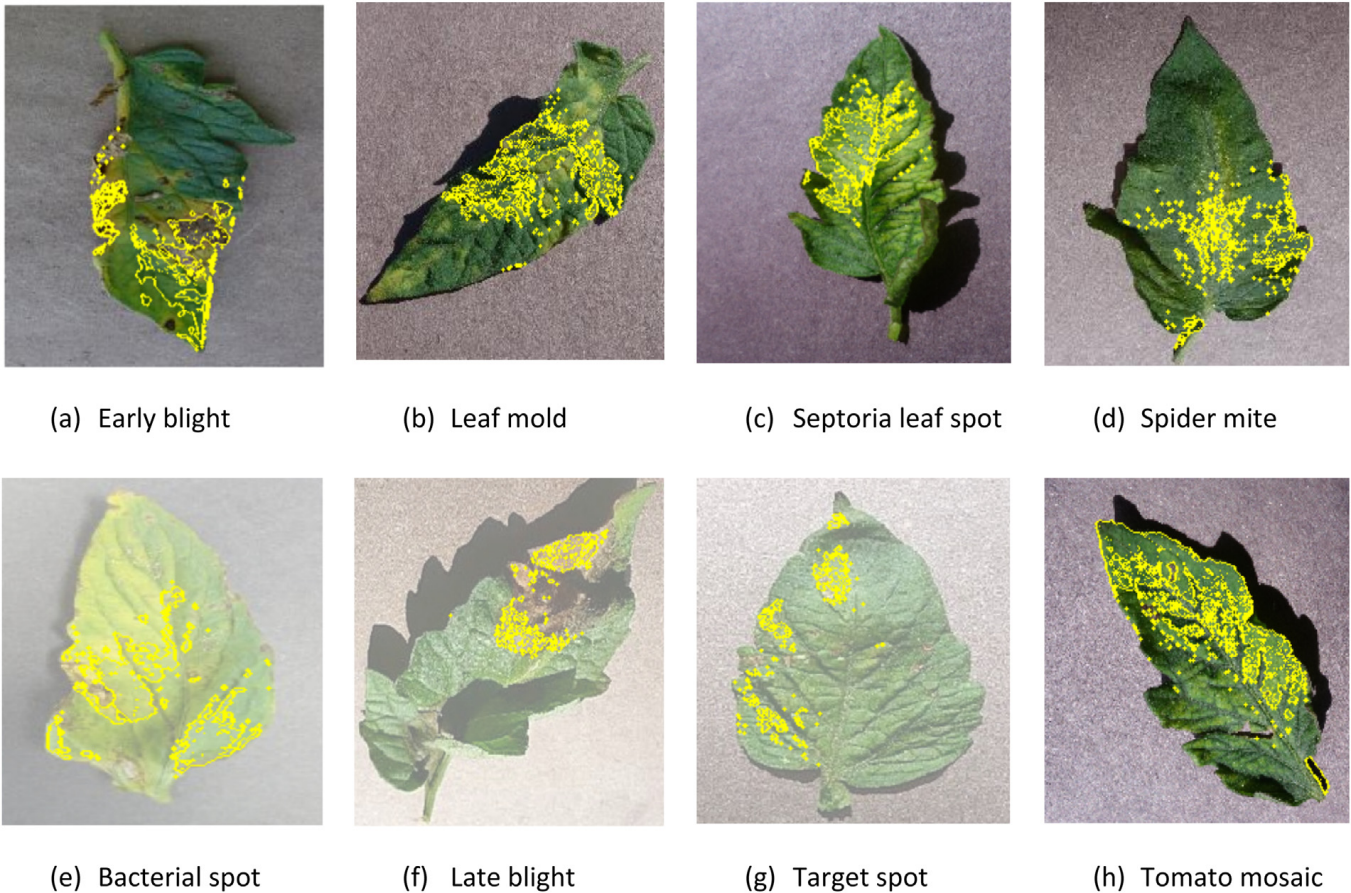
LIME explanation of saliency map for the eight classes.

We employed widely used two XAI methods, LIME and SHAP, to produce localized and comprehensive explanations for the predictions made by the proposed model on both the test and validation datasets. The LIME interpretation of a sample image from our dataset is shown in Figure X. The key parts of the input image that had a major impact on the proposed model's prediction for a particular tomato disease are highlighted in the LIME-generated illustration. LIME's local interpretable model, which provides insights into the features guiding the classification, successfully captures the decision boundaries within this instance. This representation contributes to increased transparency and makes it easier to evaluate predictions by helping to understand the behavior of the model on an individual level. Considering the first images of early blight in Fig. 5 (a), the leaf contains the early blight. The yellow overlay represents the portions of the image that have the largest impact on the model's classification decision and LIME accurately partitions the sections properly. When analyzing the LIME output for leaf mold in Fig. 5 (b), the central part of the leaf has a significant impact on the classification (Fig. 12).

**Fig. 13:**
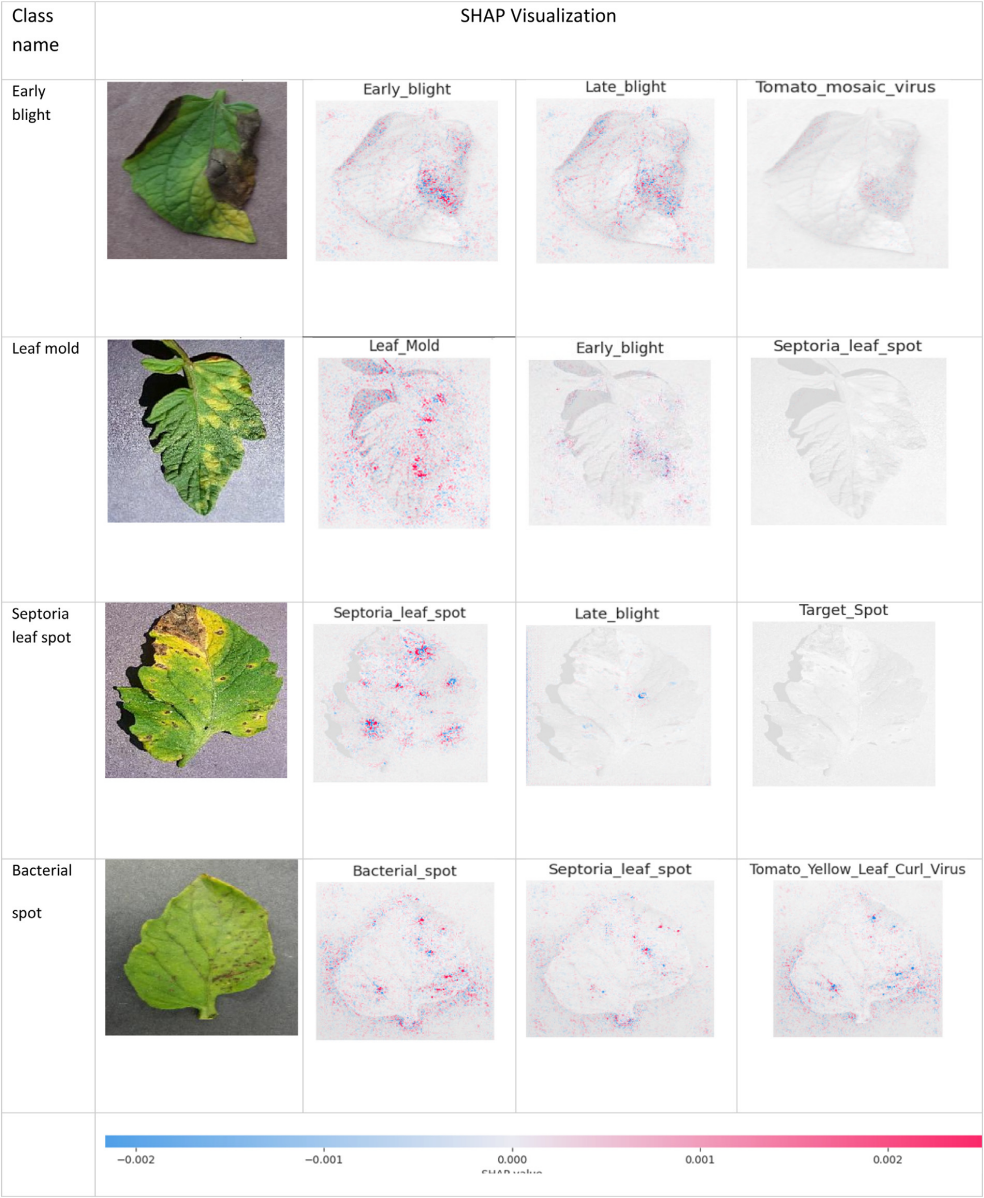
Four classes of sample images along with their respective SHAP explanation images.

Fig. 13 displays four saliency maps with varying SHAP value ranges, encompassing both negative and positive values. The SHAP values that are negative are depicted using blue pixels. These blue pixels indicate the image pixels that have a negative impact on the model's predictions. The red pixels in the image represent the positive SHAP values, indicating the pixels that contribute positively to the model predictions. We generated visual representations of the values for SHAP to illustrate the specific regions within the image that had the significantly influenced on the model's prediction. The visual representations provide a significant understanding of the model's reasoning mechanism and may aid professionals in interpreting the model's forecasting.

The SHAP results for a specific image provide explanatory visuals for four classes (Early blight, Leaf mold, Septoria leaf spot, and Bacterial spot) in Fig. 8. Based on the first row, it is visible that the first explanation for the image contains a higher number of red pixels, indicating that the leaf is affected by Early blight. On the other hand, the lack of blue pixels in late blight and Tomato mosaic virus SHAP explanation images, indicate that the input image does not depict late blight or Tomato mosaic virus. However, in the second row, the SHAP explanation images of early blight and Septoria leaf spot lack red pixels, whereas the SHAP explanation image of leaf mold contains a significant number of red pixels. This indicates that the image corresponds to leaf mold. Likewise, the SHAP explanation image for a Septoria leaf spot in the third row shows a high number of red pixels., the SHAP explanation image for a bacterial speck in the fourth row shows a notable concentration of red pixels.

#### Grad cam and Grad CAM++ visualization result

Grad-CAM analyzes each neuron for a particular decision using the gradient knowledge from the proposed model's final convolutional layer. The column labeled 'Input Image' in our results demonstrates the original images of plant leaves impacted by various diseases such as Late Blight, Tomato Yellow Leaf Curl Virus, and Septoria Leaf Spot. The Grad-CAM column displays heatmaps superimposed on the input images, emphasizing the specific regions utilized by the proposed model to detect the disease in Table 9. These heatmaps use warmer colors (reds and yellows) to show areas of higher importance and cooler colors (blues) for areas of lower importance. According to the findings from Grad-CAM++ and Grad-CAM, the left section of the Late blight image seems to be more contagious, and the corresponding heatmap has been shown accordingly in Table 9. The results of Grad CAM for Tomato Yellow Leaf Curl Virus show that the central region of the leaf has the most significant influence on classification, and the heatmap is positioned in the center section. On the other hand, when it comes to Septoria Leaf Spot, the right section of the image appears to be more infectious, and the corresponding heatmap has been displayed accordingly.

**Table 9:** Grad-CAM and Grad-CAM++ techniques visualization.

Grad-CAM++ is a more advanced version of Grad-CAM designed to provide better visual explanations for Transfer learning-based models. It solves Grad-CAM's limitations by recording more fine-grained significance weights. This is especially noticeable when there are several occurrences of the same class in an image, as well as complicated background signals. The column labeled 'Grad-CAM++' displays these enhanced heatmaps, which frequently reveal a more focused and localized activation correlating to the major aspects of the leaf disease symptoms than the original Grad-CAM

In comparing various tomato leaf disease classification models, diverse techniques were observed. Our proposed approach achieved the highest accuracy of 99.11% on a dataset of 32,535 samples across 11 classes. Notably, we incorporated XAI methods like Grad-CAM, Grad-CAM++, LIME, and SHAP for enhanced model interpretability, marking a significant advancement in transparent AI practices for agricultural applications. This outperformed other benchmarks, emphasizing the effectiveness of our method in accurately classifying tomato leaf diseases while ensuring transparency in model decision-making processes (see Table 10).

**Table 10:** Result comparisons with related studies.

Author Name	Used Architecture	Best Model (Accuracy)	Size of Dataset	Total Classes	Accuracy	XAI Used	Limitation
Al-Shamasneh et al. [6]	SVM, Random forest,	SVM	PlantVillage (18,200 images)	**10**	98.80%	**No**	Lower accuracy
Sasikaladevi Natarajan et al. [7]	Deep Fused CNN and customized KNN	KNN	PlantVillage (163,000 images)	38	99.95%	Grad-CAM and Occlusion Sensitivity Analysis	High computational complexity
Bhandari et al. [9]	EfficientNetB5	EfficientNetB5	11,000	10	99.84%	GradCAM and LIME	The data size is smaller
Paul et al. [10]	VGG-19, VGG-16, and custom model.	Custom model	32,535	11	95%	No	Lower accuracy
A. Nag et al. [11]	ResNet-50, SqueezeNet-1.1, AlexNet, VGG19, and DenseNet-121	DenseNet-121	18,160	3	99.85%.	No	No deployment, Lower size of data, Lower number of class.
Rahman et al. [12]	Support Vector Machine	Support Vector Machine	—	4	Healthy Leaf:100%, Early Blight: 95%, Septoria Leaf Spot: 90% and Late Blight: 85%	No	No deployment, Lower number of class, Single model applied.
Shoaib et al. [13]	InceptionNet1, InceptionNet2, and InceptionNet3	InceptionNet1	18,161	10	(Binary: 99.95%, six segmented: 99.12%)	Grad-CAM, Grad-CAM++	No deployment, Lower size of data.
Tarek et al. [14]	MobileNetV1, MobileNetV2, MobileNetV3 Large, MobileNetV3 Small, InceptionV3, ResNet50, AlexNet,	MobileNetV3 Large	16,004	10	99.81%	No	No deployment, Lower size of data.
Bhujel et al. [15]	Lightweight Resnet20, Lightweight model with CBAM, Lightweight model with SE attention module, SA-based model, and DA-based model.	Lightweight model with CBAM	19,510	10	99.69%	No	No deployment, Lower size of data.
Zhou et al. [16]	Deep CNN, ResNet50, DenseNet121, Restructured Residual Dense Network (RRDN)	RRDN (95%)	13,185	9	95%	No	Lower accuracy, the data size is smaller, No deployment.
Ahmad et al. [17]	ResNet, VGG16, VGG19, and Inception V3	Inception V3	Laboratory: 2364 Field: 15,216	Laboratory based: 4 Field based: 6	(Laboratory based: 99.60 Field based: 93.70)	No	the data size is smaller, No deployment.
Salih et al. [18]	CNN	CNN	6202	6	96.43%	No	No deployment, Single model utilized, the data size is smaller
Batool et al. [19]	kNN, AlexNet model	AlexNet model.	450	—	76.1%	No	No deployment, the data size is smaller, Lower accuracy
Guerrero-Ibañez et al. [20]	ResNet, VGG16Net, Inception-v3-Net, AlexNet,Proposed convolutional neural networks	Proposed convolutional neural networks	13,500	10	99%	No	No deployment, Lower size of data.
M. Li et al. [21]	ResNet50, GoogleNet, InceptionResNetV2, MobileNetV2, LMBRNet.	LMBRNet	8000	–	99.7%	No	No deployment, Lower size of data.
Our apporach	MobileNet, VGG19, Fine-Tuned EfficientNet-B0	Proposed model	32,535	11	99.11%	Grad-CAM, Grad-CAM++, LIME AND SHAP	-

### Deployment

A crucial step in making the benefits of the tomato leaf disease classification model available to end users is the model's implementation. This section outlines the deployment process, integration with a Flask web application, and how users can interact with the model for disease prediction.

#### Flask web application

**Fig. 14:**
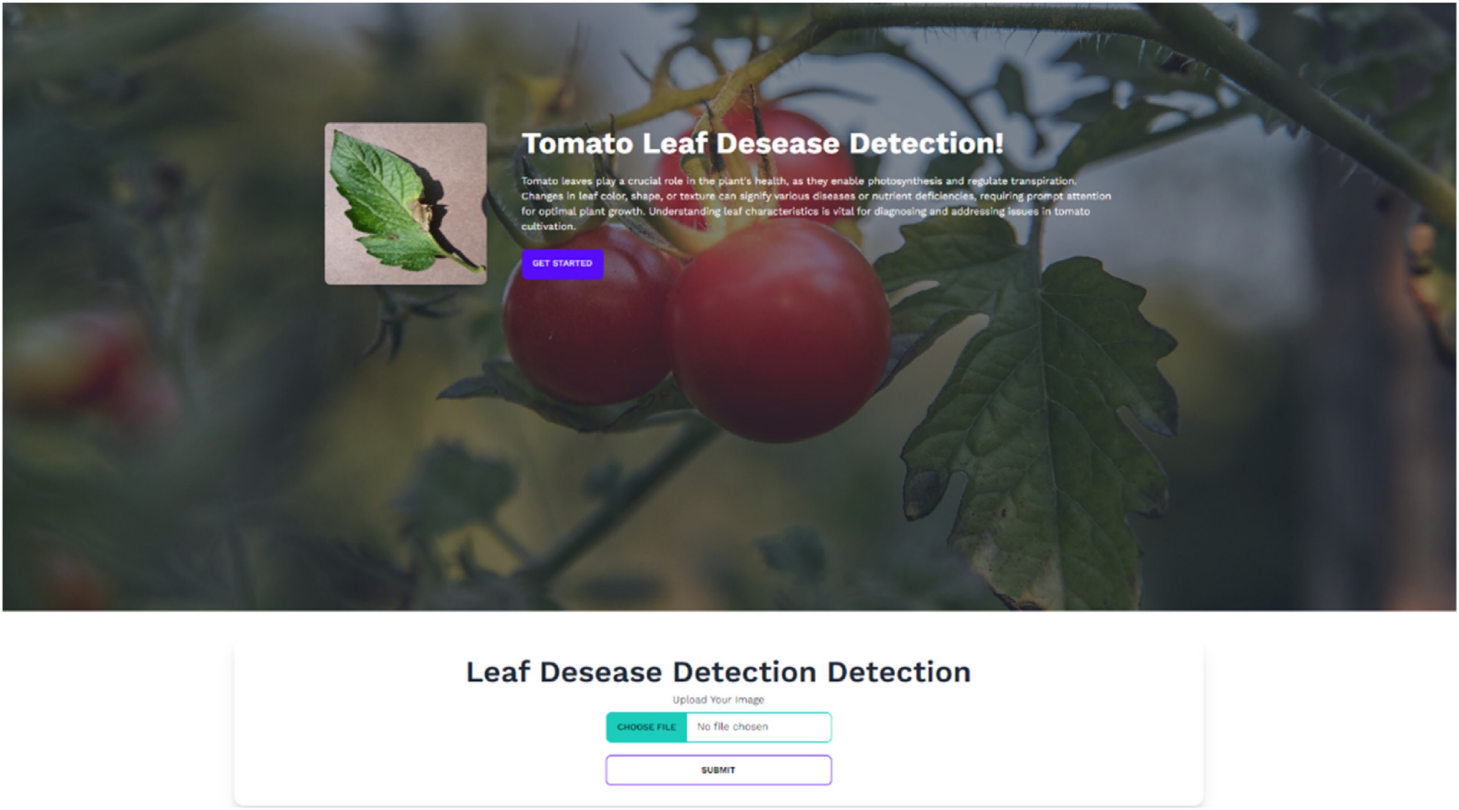
Visualization of flask web application.

The deployed system utilizes a Flask web application, allowing users to upload images of tomato leaves and receive predictions and disease solutions about the presence of diseases. The Flask application serves as the interface between users and the trained model. Users can interact with the application through a user-friendly interface, making it accessible even to those without technical expertise (See Fig. 14).

**Fig. 15:**
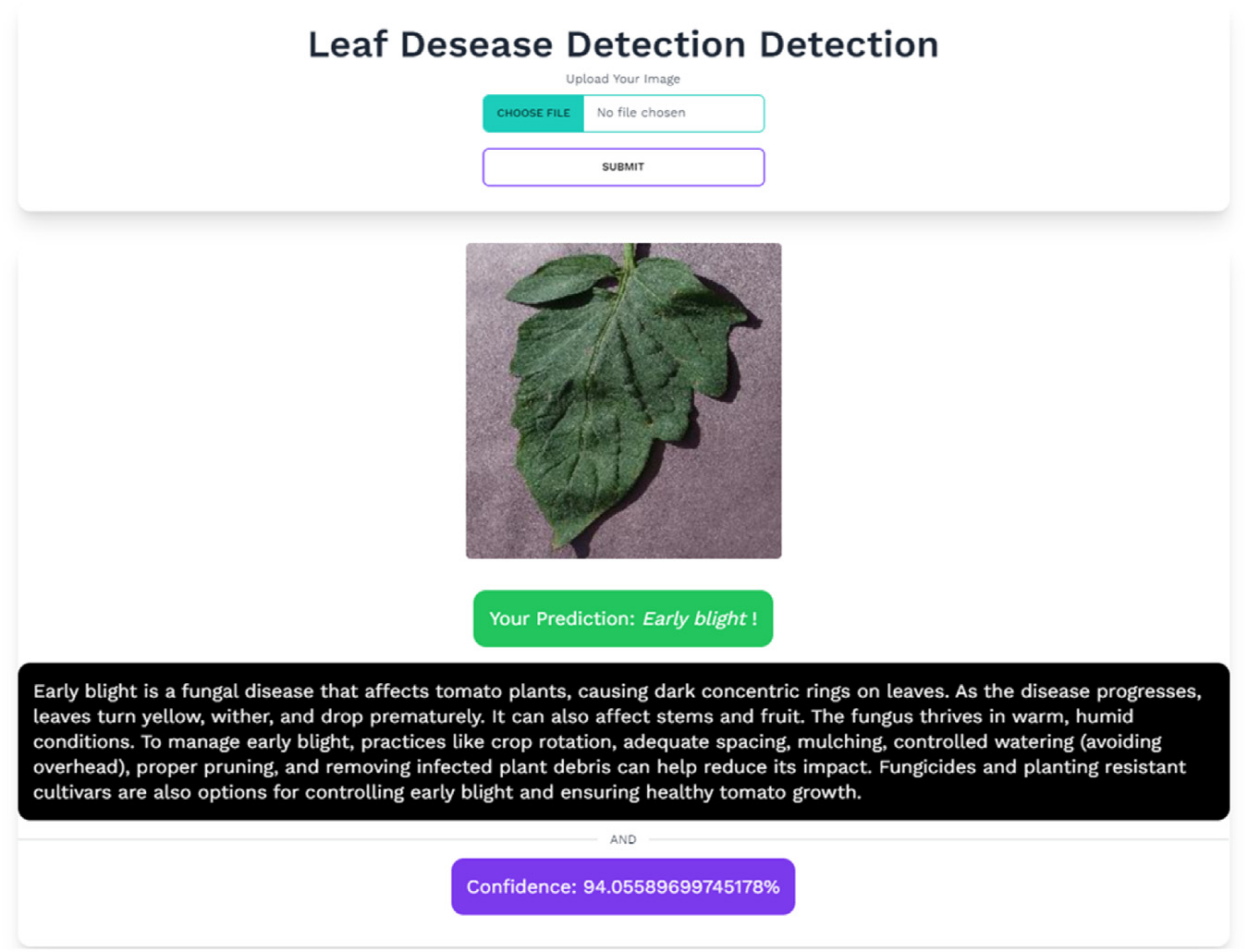
Visualization of deployment result and given solution from flask app.

The deployment phase is a critical step in implementing the model for user accessibility and utilization. The deployed system is anchored by a robust Flask web application, strategically designed to provide users with an intuitive and user-friendly interface. This interface facilitates the submission of tomato leaf images and retrieval of predictions along with corresponding disease solutions. The system's foundation lies in the well-established EfficientNet-B0 architecture, meticulously fine-tuned to proficiently classify 11 distinct tomato leaf diseases. This specialized classifier excels in capturing nuanced, disease-specific features, ensuring precision in its predictive capabilities. The user interaction with the application is seamless, catering to individuals with varying levels of technical proficiency. Users can effortlessly upload images and receive insightful predictions. Upon image submission, the model's predictive engine is initiated. The uploaded image undergoes a preprocessing journey mirroring the training process, and the model generates a comprehensive output. This output includes the predicted disease class label, a confidence score, and a detailed description of the anticipated disease solution, as illustrated in Fig. 15. To extend the accessibility of the model, a Web application has been developed. Utilizing the top-performing model saved in the "h5″ format, this application extends the system's usability and availability. For more insight and access to implementation code, the extensive resources available within the GitHub repository are explored [38].

In the spirit of transparency, the application integrates class-specific information for each disease, offering users a deeper understanding through unique names and succinct descriptions. The emphasis on user experience is evident in the application's design, facilitating effortless navigation, real-time predictions, and the storage of uploaded images and predictions for future reference. The immediacy of result generation, accomplished within seconds of image upload, significantly elevates user engagement and satisfaction levels with the Flask web application. In essence, the system not only exemplifies technical prowess in disease classification but also prioritizes a positive and enriching user experience.

## Conclusion

Tomato leaf diseases should be promptly identified and treated to bolster plant productivity, effectiveness, and quality, as incorrect diagnoses by farmers can lead to inadequate treatments, adversely affecting both plants and agroecosystems. Thus, accurate disease detection is vital. In this study, we employed transfer learning-based techniques such as EfficientNet-B0 to classify tomato leaf diseases. Our fine-tuned proposed model incorporates feature extraction and a custom classifier design. The optimization of this model was achieved using methods like Stochastic Gradient Descent (SGD) or Adam, with the latter being employed to adjust distinct learning rates for various model components, including the stem, blocks, head, and classifier. This robust and comprehensive dataset laid the groundwork for training an expert, tailored proposed model, enabling precise classification of tomato leaf diseases and ensuring adaptability across diverse datasets. Additionally, we integrated Explainable Artificial Intelligence (X-AI) practices, including Grad-CAM, Grad-CAM++, LIME, and SHAP to improve the interpretability of our model, providing visual insights into its decision-making process. This implementation resulted in our model achieving an impressive 99% accuracy and recall rate, the highest among all models, further bolstered by the incorporation of data augmentation techniques.

Future studies may broaden disease detection beyond tomato leaves to encompass a wider range of plant illnesses, promoting more thorough agricultural disease management. A diverse approach to plant health monitoring is also provided by the incorporation of sensors or smart appliances that may evaluate fruit quality and maturity. The field of agricultural technology will improve as a result of research into the effects on the environment, real-time monitoring, data integration, automation, user-friendly tools, machine learning advancements, collaborative efforts, education, and outreach. Through creative and affordable solutions, these initiatives seek to improve crop health, environmental sustainability, and farmers' capacity to support their families.

## Limitations

This study is limited to the detection of diseases in tomato leaves, thus leaving other crops and plant diseases unexplored. The model could also be less generalizable to real-world agricultural settings because of its reliance on existing datasets. High costs and limited access to advanced technologies such as sensors and smart devices may challenge the adoption of these technologies by small-scale farmers.

## Ethics statements

The research uses publicly available datasets, ensuring that all ethical guidelines regarding data usage policies are followed. No sensitive or personal information is involved, hence participant confidentiality and integrity are maintained.

## Supplementary material *and/or* additional information [OPTIONAL]

You may also submit supplementary material with your article. This is not compulsory. If you do submit supplementary files, you are welcome to provide supporting details in this OPTIONAL section. More information is available in the Guide for Authors.

## CRediT authorship contribution statement

**Md Assaduzzaman:** Investigation, Methodology, Resources, Writing – original draft, Writing – review & editing. **Prayma Bishshash:** Conceptualization, Validation, Writing – original draft, Formal analysis. **Md. Asraful Sharker Nirob:** Formal analysis, Investigation, Visualization, Writing – original draft. **Ahmed Al Marouf:** Formal analysis, Resources, Supervision. **Jon G. Rokne:** Formal analysis, Supervision. **Reda Alhajj:** Writing – original draft, Resources, Supervision.

## Declaration of competing interest

The authors declare that they have no known competing financial interests or personal relationships that could have appeared to influence the work reported in this paper.

## Data Availability

Data will be made available on request.
